# Economic costs attributable to modifiable risk factors: an analysis of 24 million urban residents in China

**DOI:** 10.1186/s12916-024-03772-7

**Published:** 2024-11-21

**Authors:** Xuechen Xiong, Zhaohua Huo, Yinan Zhou, David M. Bishai, Karen A. Grépin, Philip M. Clarke, Cynthia Chen, Li Luo, Jianchao Quan

**Affiliations:** 1 School of Public Health, LKS Faculty of Medicine, The University of Hong Kong, Hong Kong SAR, China; 2 Department of Psychiatry, Faculty of Medicine, The Chinese University of Hong Kong, Hong Kong SAR, China; 3 School of Public Health, Fudan University, Shanghai, China; 4 Health Economics Research Centre, Nuffield Department of Population Health, University of Oxford, Oxford, UK; 5 Saw Swee Hock School of Public Health, National University of Singapore, Singapore, Singapore; 6 HKU Business School, The University of Hong Kong, Hong Kong SAR, China

**Keywords:** Healthcare cost, Productivity loss, Societal cost, Modifiable risk factors, Urban China

## Abstract

**Background:**

Estimating the economic burden of modifiable risk factors is crucial for allocating scarce healthcare resources to improve population health. We quantified the economic burden attributable to modifiable risk factors in an urban area of China.

**Methods:**

Our Shanghai Municipal Health Commission dataset covered 2.2 million inpatient admissions for adults (age ≥ 20) in public and private hospitals in 2015 (1,327,187 admissions) and 2020 (837,482 admissions). We used a prevalence-based cost-of-illness approach by applying population attributable fraction (PAF) estimates for each modifiable risk factor from the Global Burden of Diseases Study (GBD) to estimate attributable costs. We adopted a societal perspective for cost estimates, comprising direct healthcare costs and productivity losses from absenteeism and premature mortality. Future costs were discounted at 3% and adjusted to 2020 prices.

**Results:**

In 2020, the total societal cost attributable to modifiable risk factors in Shanghai was US$7.9 billion (95% uncertainty interval [UI]: 4.6–12.4b), mostly from productivity losses (67.9%). Two health conditions constituted most of the attributable societal cost: cancer (51.6% [30.2–60.2]) and cardiovascular disease (31.2% [24.6–50.7]). Three modifiable risk factors accounted for half of the total attributable societal cost: tobacco (23.7% [16.4–30.5]), alcohol (13.3% [8.2–19.7]), and dietary risks (12.2% [7.5–17.7]). The economic burden varied by age and sex; most of the societal costs were from males (77.7%), primarily driven by their tobacco and alcohol use. The largest contributor to societal costs was alcohol for age 20–44, and tobacco for age 45 + . Despite the COVID-19 pandemic, the pattern of major modifiable risk factors remained stable from 2015 to 2020 albeit with notable increases in attributable healthcare costs from cancers and productivity losses from cardiovascular diseases.

**Conclusions:**

The substantial economic burden of diseases attributable to modifiable risk factors necessitates targeted policy interventions. Priority areas are reducing tobacco and alcohol consumption and improving dietary habits that together constitute half of the total attributable costs. Tailored interventions targeting specific age and sex groups are crucial; namely tobacco in middle-aged/older males and alcohol in younger males.

**Supplementary Information:**

The online version contains supplementary material available at 10.1186/s12916-024-03772-7.

## Background

Reducing the burden of modifiable risk factors has long been championed in public health policy to improve population health. Globally, almost half (47.8%) of all disability-adjusted life-years (DALYs) were attributed to modifiable risk factors; with high systolic blood pressure and tobacco as the leading risk factors for mortality in 2019 [1]. Other major modifiable environmental, occupational, behavioral, and metabolic risk factors include high body-mass index, physical inactivity, alcohol consumption, and poor diet [2–4].

From 2010 to 2019, the largest declines in exposure to harmful risks globally were those strongly linked to social and economic development, including household air pollution; unsafe water, sanitation, and handwashing; and child growth failure [5]. However, the pattern of attributable risk factors varied widely by geography [6]. China initiated a new round of healthcare system reform in 2009 with the goal of providing equal and guaranteed essential medical and health services for all by 2020 [7]. Significant achievements include universal coverage of social health insurance and the establishment of an essential medicine system [8]. Moreover, the country has rapidly urbanized with the proportion of urban residents climbing from 18% in 1978 to nearly 65% in 2020 [9], and is forecasted to continue rising [10]. China is undergoing rapid epidemiological shifts transforming population health and the pattern of risk factor exposure [11–13].

Past studies in China have focused on the burden of specific conditions such as cancer and cardiovascular disease [14–17], or specific risk factors such as tobacco [18, 19]. Yet, there is little evidence of the relative effects attributable to multiple modifiable risk factors. Tracking the health and economic burden attributable to modifiable risk exposure can help policymakers prioritize public health actions and focus attention on the risk factors with the biggest potential gains. This study aims to estimate the economic burden of modifiable risk factors in Shanghai, an urban area in China, and the relative contributions of the multitude of modifiable risk factors.

## Methods

### Study design

We estimated the economic cost of health conditions attributable to modifiable risk factors using a prevalence-based cost-of-illness approach [20]. We adopted a societal perspective for cost estimates comprising of the direct costs from healthcare use and the indirect costs from productivity losses due to absenteeism and premature mortality (defined as death before life expectancy). We estimated healthcare costs and productivity losses attributable to modifiable risk factors using the population attributable fraction (PAF) for every health condition and risk factor pair [2, 6]. PAF is the proportion of the prevalent health burden attributable to each risk factor calculated from the relative risks and population exposure (Additional file 1: Text S1) [21, 22]. Where a disease has multiple risk factors, the PAF may overestimate the impact of each risk factor if cases with multiple risk factors are not mutually exclusive. To adjust for this overlap, we applied the Krueger approach [23, 24] used to combine risk factors in many previous studies (Additional file 1: Text S1) [24–27]. We examined 20 risk factors (level 2 risks from the Global Burden of Disease (GBD) comparative risk assessment framework) commonly used in other studies as they include most modifiable risk factors (Additional file 1: Table S1) [26, 28, 29]. We examined 22 mutually exhaustive health conditions (level 2 causes with 22 disease and injury aggregate groupings in GBD).

### Setting

Our study data covered Shanghai, one of the largest and most economically developed urban areas in China, with over 24 million permanent residents. Total annual health expenditure in Shanghai amounted to RMB 152 billion (US$22b) in 2015 and RMB 263 bn (US$38b) in 2020, accounting for 5.7% and 6.7% of Shanghai's gross domestic product (GDP) respectively (per capita expenditure of RMB 6,362 [US$936] in 2015 and RMB 10,592 [US$1,558] in 2020) [30]. Healthcare expenditures in Shanghai were largely funded by social contributions (56.8%), government support (22.3%), and out-of-pocket payments (20.9%) in 2020 [30]. The coverage rate of social medical insurance exceeds 97% in Shanghai [31]. Healthcare services in Shanghai are mainly provided by the public sector, which financed 94% in 2015 and 88% in 2020 of total health expenditures [30]. Public providers dominate hospital services in Shanghai; providing 91.2% of outpatient services, 93.4% of inpatient services, and 95.8% of surgical services in 2020 [31]. Hospitals, specifically secondary and tertiary medical institutions, represented 68% of Shanghai’s total health expenditures in 2020, amounting to RMB 179 billion (US$26b), of which 56% was spent on inpatient care (38% of all health expenditures; RMB 100 billion, US$15b).

### Data sources

We analyzed data from the Shanghai Municipal Health Commission covering all inpatients aged 20 and over in both public and private hospitals in 2015 (1,327,187 admissions) and 2020 (837,482 admissions). Data include health conditions, length of stay, medical expenses during hospitalization (medical treatment, surgery, diagnostics, nursing, rehabilitation, physiotherapy, drugs, blood products, and consumables), and discharge status (cured, improved, not cured, died, others). We used the principal diagnosis codes in hospitalization records to classify health conditions according to the WHO International Classification of Diseases, 10th Revision (ICD-10) codes (Additional file 1: Table S2). We extracted the modifiable risk factor-specific attributable disability-adjusted life-years (DALYs) for each health condition from GBD 2020 and 2015 data [32]. PAF estimates for each modifiable risk factor were determined by dividing risk factor-specific attributable DALYs by the total DALYs caused by all risk factors for each health condition.

The income distribution of urban Chinese residents was derived from the Chinese Household Income Project (CHIP) 2018 [33], which used a stratified multi-stage sampling method to survey 71,266 individuals nationwide (36,259 from urban areas). We proxied income distribution of Shanghai residents by age and sex from data on urban residents in the CHIP 2018 survey standardized to the mean income level for Shanghai residents from the Shanghai Statistical Yearbook [30] (Additional file 1: Table S3). The income growth rate was the annualized change in real annual salary of workers in Shanghai from 2008 to 2021 at 10% in nominal terms (Additional file 1: Table S4) [30]. The age-and sex-specific labor force participation rates and mortality rates of residents in Shanghai were derived from the Shanghai Population Census Yearbook 2020, a comprehensive survey for the seventh national population census (Additional file 1: Table S5-6) [34].

### Cost estimates

We adopted a societal perspective for cost estimates comprising healthcare costs and productivity losses (formulas to estimate costs in Additional file 1: Text 2). Healthcare costs were calculated based on the overall economic cost-to-charge ratio of hospitalizations, which stood at 0.95 in 2015 and 1.05 in 2020 [35, 36]. These ratios indicate healthcare charges closely align with the economic cost, given that most inpatient admissions (95%) were treated at non-profit public hospitals in the study years. The calculated PAFs for each pairing of health condition and risk factor were multiplied by the inpatient expenditure to estimate the attributable healthcare cost for each modifiable risk factor.

Productivity losses were composed of absenteeism-related income loss during inpatient hospitalization and human capital loss from premature mortality. Income losses from absenteeism were estimated by multiplying the age- and sex-specific daily wage rates and labor force participation rates with length of hospital stay. Human capital loss from premature mortality was estimated as the total expected future earnings, calculated as the present value of earnings by age and sex from their age at death until life expectancy (male: 81 years, female: 86 years) [30], multiplied by the age- and sex-specific labor force participation rates. Income data for age over 80 years were unavailable, thus earnings were curtailed at 80 years for both males and females. To estimate the number of premature deaths in Shanghai, we applied the distribution of deaths by health conditions in hospitals by age and sex to the age-sex specific mortality rate of residents in Shanghai (Additional file 1: Table S6). Thus, we assumed that the distribution of deaths by health condition for each sex-specific 5-year age group mirrored that of the matching age-sex group observed in the hospital setting. PAFs were multiplied by the productivity loss to estimate attributable productivity loss for each modifiable risk factor.

We used an annual discount rate of 3% reflecting the real inflation rate of both healthcare services and general price inflation in Shanghai measured by the consumer price indices (Additional file 1: Table S7) [35]. We estimated the attributable economic burden for 27 separate age-sex groups (20–24, …, 75–79, 80 and above). For reporting purposes, we categorized results into three broad age groups (20 to 44, 45 to 64, and 65 and above). Cost estimates are reported in US dollars (USD) using a consistent USD-to-RMB exchange rate of 6.8.

### Uncertainty estimates and sensitivity analyses

To capture the uncertainty in estimates, we simulated the mean and uncertainty intervals (UIs) using independent 1000 draws of the PAFs from a beta distribution with parameters from the GBD 2020. The GBD 2020 study provided UIs for the attributable DALYs of modifiable risk factors for each health condition. Since the PAF is the proportion of DALYs attributed to a specific risk factor out of the total DALYs caused by all risk factors, we simulated the PAF of modifiable risk factors for each health condition. We used the 2.5 and 97.5 percentiles of the draws as lower and upper uncertainty estimates for each estimate. Analyses were conducted using R version 4.4.

We conducted sensitivity analyses to assess uncertainties in the long-term economic outlook by exploring scenarios of (1) increasing the wage rate to regional advanced economies; (2) lowering the wage growth rate from 10 to 5%; (3) varying the discount rate from 0% (undiscounted) to 5% compared to the base case of 3%. Additional sensitivity analyses were conducted to assess labor market assumptions using retirement age instead of life expectancy and exploring recent policy reforms to gradually increase retirement age by 2040. In these analyses, we assumed that individuals have no formal economic output (zero participation/wages) at ages above the retirement age in these scenarios: (1) current retirement age of 60 years for males and 55 years for females; (2) policy reform to 63 years for males and 58 years for females by 2040; (3) 65 years for males and 60 years for females.

## Results

Inpatient admissions with a principal diagnosis of the 22 health conditions fell from 1,327,187 in 2015 to 837,482 in 2020; hospital admissions by age and sex shown in Additional file 1: Table S8. Admissions were evenly distributed across age groups. In 2020, female inpatients slightly outnumbered males, comprising 56% of admissions but only 47% of healthcare expenditure and 38% of deaths in hospital (Additional file 1: Table S6, S9).

### Attributable healthcare costs

**Fig. 1:**
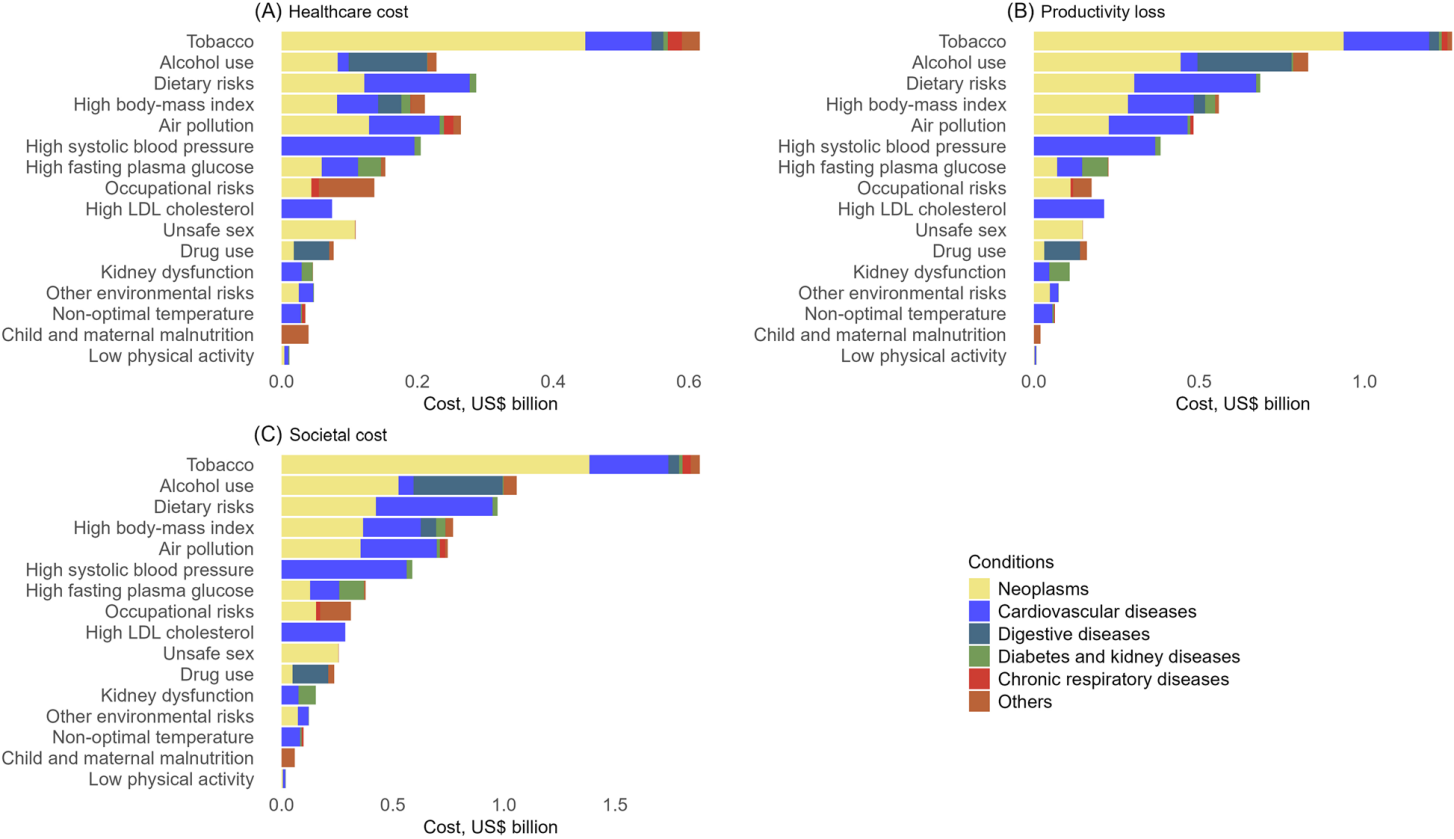
Attributable healthcare cost, productivity loss and societal cost by modifiable risk factor in 2020

We estimated that inpatient healthcare costs of US$2.4 billion (95% UI: 1.3–3.9b) in 2020 was attributable to the modifiable risk factors. Two conditions accounted for most of the cost: cancer (48.4% [26.1–58.8]) and cardiovascular disease (30.1% [23–47.8]) (Table 1, Fig. 1). The most important risk factors for cancers were tobacco (39.9% [27.5–49.7]), air pollution (11.5% [5.2–19.3]) and dietary risks (10.9% [4.5–19.8]). For cardiovascular diseases, the most important risk factors were high systolic blood pressure (23.3% [17.8–29.6]), dietary risks (18.4% [13.4–24]), and air pollution (12.3% [9–16]) (Additional file 1: Table S10).

**Table 1:** Healthcare cost, productivity loss, and societal cost attributable to modifiable risk factors by health condition in 2015 and 2020

Health Conditions	Healthcare cost		Productivity loss		Societal cost	
	US$ million [UI]	% [UI]	US$ million [UI]	% [UI]	US$ million [UI]	% [UI]
**Year 2015**						
All conditions	2,413 [1,366–4,083]	100%	3,523 [1,879–6,084]	100%	5,936 [3,245–9,987]	100%
Cardiovascular diseases	950 [568–1,444]	37% [27–52]	669 [427–962]	15.8% [11.4–34.6]	1,619 [995–2,406]	24.1% [18.3–42.4]
Chronic respiratory diseases	155 [101–215]	5.5% [4.4–9.7]	92 [38–158]	2.6% [1.2–5.5]	247 [140–373]	3.7% [2.3–7.3]
Diabetes and kidney diseases	145 [85–224]	5.7% [3.5–9.4]	93 [46–157]	2.6% [1.4–5.2]	238 [131–381]	3.8% [2.3–6.9]
Digestive diseases	264 [120–451]	11.6% [5–17.8]	232 [130–340]	5.6% [3.8–12.2]	496 [250–791]	7.9% [4.6–15.1]
Maternal and neonatal disorders	186 [186–186]	4.8% [6.4–10.2]	45 [45–45]	0.7% [0.9–2.2]	231 [231–231]	2.3% [3–5.8]
Musculoskeletal disorders	31 [5–63]	1.6% [0.2–2.6]	9 [2–15]	0.2% [0.1–0.5]	39 [7–78]	0.8% [0.2–1.4]
Neoplasms	513 [249–972]	24.9% [9.1–34.2]	2,004 [895–3,937]	64.7% [34.3–71.7]	2,517 [1,144–4,909]	49.2% [20.9–56.8]
Neurological disorders	35 [1–100]	2.6% [0.2–3.6]	47 [25–74]	1.2% [0.7–2.6]	82 [26–174]	1.7% [0.3–2.9]
Sense organ diseases	97 [25–196]	5% [1.3–8]	3 [1–4]	0.1% [0–0.2]	100 [25–200]	2% [0.5–3.5]
Others	36 [24–51]	1.3% [1–2.3]	330 [270–392]	6.4% [6.5–16.7]	366 [295–443]	4.4% [4.5–9.2]
**Year 2020**						
All conditions	2,541 [1,408–4,083]	100%	5,389 [3,181–8,343]	100%	7,939 [4,589–12,426]	100%
Cardiovascular diseases	842 [542–1,227]	30.1% [23–47.8]	1,906 [1,272–2,651]	31.8% [25.6–50]	2,748 [1,814–3,879]	31.2% [24.6–50.7]
Chronic respiratory diseases	51[35–70]	1.7%[1.3–3.3]	37[27–48]	0.6%[0.5–1.1]	88[61–119]	1%[0.8–1.8]
Diabetes and kidney diseases	99[65–141]	3.4%[2.5–6.3]	224[141–337]	4%[2.5–6.8]	323[205–478]	3.8%[2.4–6.8]
Digestive diseases	219[114–341]	8.4%[4.5–14.7]	457[272–641]	7.7%[5.5–13.4]	676[386–983]	7.9%[5.3–13.5]
Maternal and neonatal disorders	39[39–39]	1%[1.2–2.3]	20[20–20]	0.2%[0.3–0.5]	59[59–59]	0.5%[0.6–1.1]
Musculoskeletal disorders	44[14–77]	1.9%[0.6–3.3]	66[30–97]	1.2%[0.7–2.1]	110[44–174]	1.4%[0.7–2.5]
Neoplasms	1,121[544–1,974]	48.4%[26.8–58.8]	2,604[1,353–4,432]	53.1%[32.4–61.3]	3,725[1,898–6,406]	51.6%[30.2–60.2]
Neurological disorders	34[10–75]	1.8%[0.3–2.9]	58[43–87]	1%[0.6–1.7]	92[53–163]	1.3%[0.6–2.1]
Sense organ diseases	79[34–121]	3%[1.6–5.6]	4[2–6]	0.1%[0–0.1]	83[36–127]	1%[0.5–1.9]
Others	13[11–16]	0.4%[0.4–0.8]	22[21–23]	0.3%[0.3–0.6]	36[32–40]	0.3%[0.3–0.6]

Other conditions include mental disorders, nutritional deficiencies, other non-communicable diseases, self-harm and interpersonal violence, substance use disorders, unintentional injuries, enteric infections, HIV/AIDS and sexually transmitted infections, respiratory infections and tuberculosis, and other infectious diseases

*US$* US dollar 2020 prices, *UI* uncertainty interval

Tobacco use accounted for the single largest share of total attributable inpatient healthcare costs in 2020 at 24.2% (16.3–31.7) amounting to US$616 million (413–805 m); mostly cancer (72.6%, 50–90.5) and cardiovascular diseases (15.8%, 11.4–20.3) (Fig. 1, Additional file 1: Table S11). Dietary risk accounted for the second highest proportion of attributable healthcare expenditures at 11.3% (6.7–17.2) at US$273 million (169–438 m), mostly cardiovascular diseases (54.1%, 39.3–70.5) and cancer (42.5%, 17.7–77.4). Air pollution accounted for the third-highest attributable healthcare expenditures, at 10.4% (5.9–15.7) or US$264 million (150–400 m), mostly cancer (48.8%, 22.2–81.8) and cardiovascular diseases (39.3%, 28.6–51).

### Attributable productivity losses

In 2020, we estimated that productivity losses of US$5.4 billion (3.2–8.3b) were attributable to the modifiable risk factors. The largest productivity losses were from cancer (53.1% [32.4–61.3]), cardiovascular diseases (31.8% [25.6–50]), and digestive diseases (7.7% [5.5–13.4]) (Table 1). The greatest risk factors for cancer were tobacco (36.0% [25.3–45]), alcohol use (17.0% [10–26.1]), and dietary risks (11.6% [5.2–19.2]) (Additional file 1: Table S10). For cardiovascular diseases, the greatest risk factors were dietary risks (19.4% [15.1–23.7]), high systolic blood pressure (19.3% [13.9–24.3]), and tobacco (13.6% [10.3–17.2]). The main risk factors for digestive diseases were alcohol use (62.4%) and drug use (23.6%).

Overall, tobacco accounted for the largest proportion of attributable productivity losses in 2020 at 23.4% (16.4–30) or US$1.3 billion (0.9–1.6b); mostly cancer (74.0%) and cardiovascular diseases (20.4%). Alcohol use accounted for the second highest attributable productivity loss (15.4%, US$0.8 billion) followed by dietary risks (12.7%, US$0.7 billion), high body-mass index (10.4%, US$0.6 billion), and air pollution (8.9%, US$0.5 billion) (Fig. 1, Additional file 1: Table S11).

### Attributable societal costs

We estimated the total societal costs of modifiable risk factors at US$8.0 billion (4.6–12.4b) in 2020. Productivity loss accounted for 67.9% of attributable societal costs at US$5.4 billion (3.2–8.3b) with US$2.5 billion (1.4–4.1b) from healthcare costs. Cancer (51.6% [30.2–60.2]) and cardiovascular disease (31.2% [24.6–50.7]) accounted for most of the attributable societal costs (Table 1). The leading risk factors for attributable societal cost were tobacco (23.7% [16.4–30.5], US$1.9 billion [1.2–3.1b]), alcohol (13.3% [8.2 – 19.7], US$1.1 billion [0.7–1.6b]), dietary risk (12.2% [7.5–17.7], US$1.0 billion [0.6–1.4b]), high body-mass index (9.7% [3–19.8], US$0.8billion [0.2–1.6b]), and air pollution (9.4% [5.5–14.1], US$0.7 billion [0.4–1.1b]) (Fig. 1).

### Age-specific and sex-specific societal costs

**Fig. 2:**
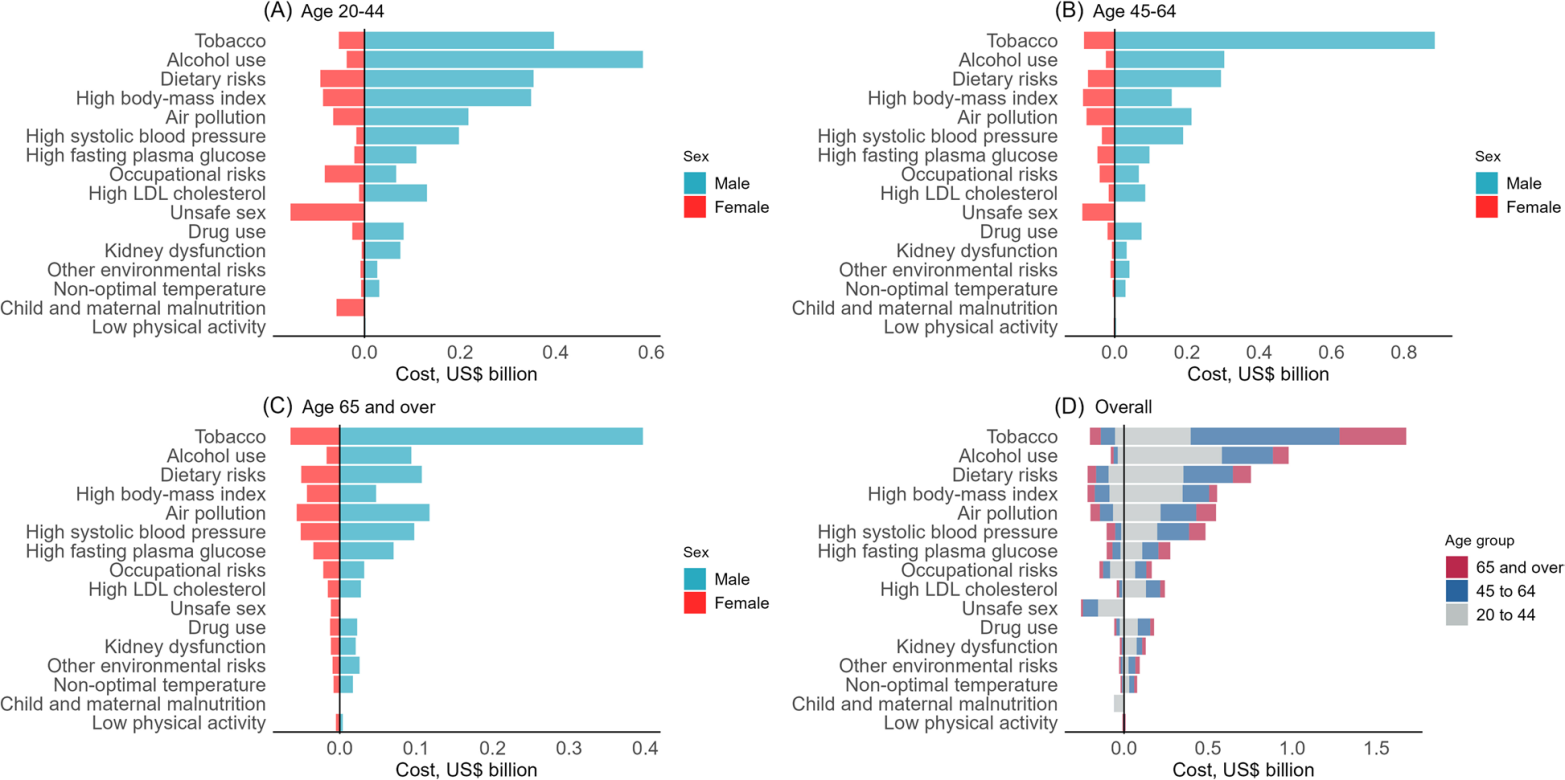
Attributable societal cost of modifiable risk factors by age and sex in 2020

Attributable societal costs varied by age and sex for risk factors (Fig. 2) and health conditions (Additional file 1: Fig. S1). Despite fewer males being hospitalized in 2020 (44% vs 56% for females), they accounted for most of the societal costs (77.7% vs 22.3% for females). In females, the main societal costs were from unsafe sex (14.4% [8.8–20.7]), high body-mass index (12.3% [2.2–25.3]), dietary risks (12.2% [6.9–19.3]), tobacco (11.5% [8.8–20.7]), and air pollution (11.3% [5.8–17.7]), while in males they were tobacco use (27.2% [19.5–33.7]), alcohol use (15.9% [10.1–22.4]), dietary risks (12.2% [7.7–17.3]), high body-mass index (9.0% [3.2–18.2]), and air population (8.9% [5.4–13.1]). Attributable societal costs were concentrated in people aged below 45 (20–44 years: 42.2%, 45–64 years: 39.0%, 65 years and over: 18.8%). Alcohol use (18.5% [12–26.2]) was the largest contributor to societal costs in those aged 20–44 years, and tobacco was the largest in people aged 45 and over (45–64 years: 31.3% [22.2–39.6]; 65 years and over (30.9% [21.4–37.4]).

### Changes in costs from 2015 to 2020

Despite a 32% decrease in the number of hospitalizations from 2015 to 2020, and a reduction in the average length of hospitalization stays from 12.8 days to 6.7 days (Additional file 1: Table S12), total healthcare costs attributable to modifiable risk factors slightly increased from US$5.9 billion (3.2–10.0b) in 2015 to US$7.9 billion (4.6–12.4b) in 2020. Across all health conditions, cancer had the largest increase in attributable healthcare costs from 24.9% in 2015 to 48.4% in 2020; while cardiovascular diseases had the largest increase in attributable productivity loss from 15.8% in 2015 to 31.8% in 2020 (Table 1).

**Fig. 3:**
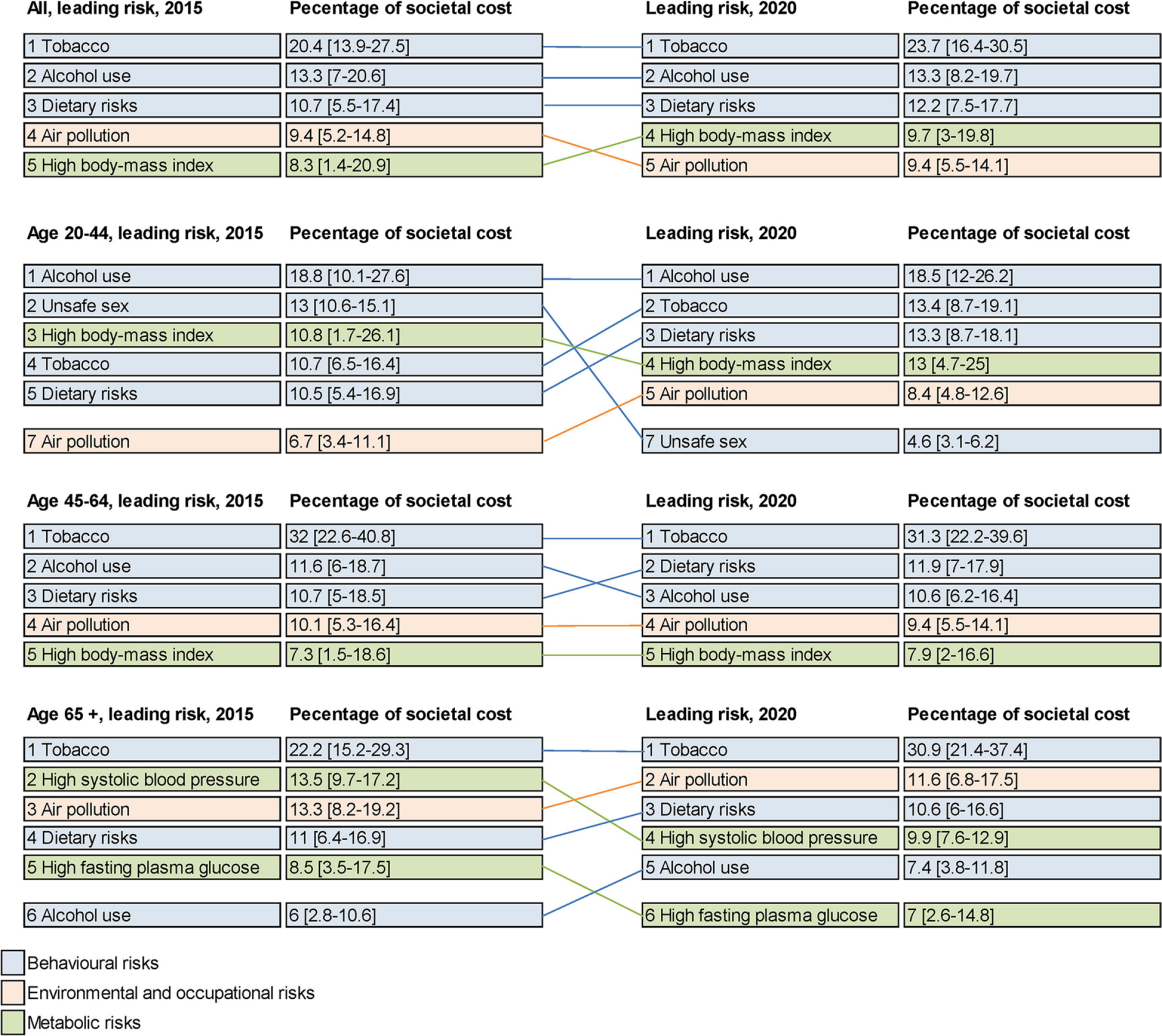
Leading risk factors by attributable societal cost, 2015–2020

The distribution of leading attributable risk factors was relatively stable from 2015 to 2020. Tobacco, alcohol and dietary risks consistently ranked as the highest three risk factors for overall attributable societal costs in 2015 and 2020 (Fig. 3). Alcohol use remained the primary risk factor contributing to societal costs for those aged 20–44 years, and tobacco use for those aged 45 and over.

### Sensitivity analyses

We ran scenarios for 2020 estimates adjusting wage rate, wage growth rates, and discount rates (Additional file 1: Table S13). Increasing the wage rate of Shanghai to that of an advanced regional economy (e.g. Singapore) would lead to an 80% increase in attributable societal costs from US$7.9 billion to US$14.6 billion, with productivity loss growing to 82% of the societal costs (Scenario 1). Halving the wage growth rate from 10 to 5% would lead to a 63% decrease in attributable societal costs, bringing it down from US$14.6 billion to US$9.1 billion with productivity loss accounting for 72% of the total (Scenario 2). Varying the discount rate gave total societal cost estimates ranging from US$7.6 billion (5% discount rate) to US$10.7 billion (undiscounted) (Scenario 3, 4).

Our analysis varying the retirement age threshold in the calculation of future income losses gave lower societal costs estimates compared to our base case using the lifespan approach, even after accounting for recent policy reforms to gradually raise the retirement ages in China (Additional file 1: Table S14). Assuming individuals earn no wages above the retirement age would result in a 34% decrease in attributable societal costs from US$7.9 billion (base case) to US$5.2 billion (of which 51% were productivity losses) at retirement ages of 60 years for males and 55 years for females; a 23% decrease in attributable societal costs to US$6.1 billion (58% from productivity losses) under the policy reforms of 63 years for males and 58 years for females; and a 17% decrease in attributable societal costs to US$6.6 billion (61% from productivity losses) at 65 years for males and 60 years for females.

## Discussion

In 2020, we estimated societal costs of US$7.9 billion attributable to modifiable risk factors in Shanghai (US$2.5 billion on inpatient healthcare, US$5.4 billion from productivity loss). Three risk factors (tobacco, alcohol, and dietary risks) accounted for half of the attributable productivity losses and societal costs; while most of the attributable inpatient healthcare costs were from four risk factors (tobacco, dietary risks, air pollution and alcohol). The economic burden of risk factors varied by age and sex with tobacco use posing the largest burden in older groups (age 45 +), and alcohol use in younger groups (age 20–44). Younger adults aged 20–44 were more affected by behavioral factors such as smoking, drinking, and poor diet; while for people aged over 45 years old, costs were driven by both behavioral factors and metabolic risks such as high body-mass index, high systolic blood pressure and high fasting plasma glucose. Despite having fewer inpatient admissions, males accounted for greater healthcare costs and productivity losses, primarily driven by tobacco use and alcohol use. The contribution of risk factors to societal costs was relatively stable from 2015 to 2020, with tobacco remaining the leading attributable cause. Cancer and cardiovascular disease continued to account for the most of costs with notable increases in healthcare costs from cancer and increases in productivity losses from cardiovascular diseases.

The attributable societal cost in urban China was mainly driven by productivity losses (67.9% vs 32.1% for healthcare costs). This was similar to recent studies in Singapore and Japan that found that productivity loss accounted approximately 70% of societal costs [26, 37], despite differences in methodology and unit cost values across these settings. The study in Japan took a broader approach by including outpatient and inpatient costs in contrast to ours and the Singapore study that focused on inpatient costs. For estimating productivity loss, the Japanese study considered long-term patient care including formal and informal care, whereas ours and the Singapore study focused on absenteeism and premature death. These studies used lifespan as the period to estimate future income losses from premature death. Although many retirees receive income from pensions, these are transfer payments that do not contribute to formal economic output. Nevertheless, a small percentage participate in the formal labor force and thus remain economically active post-retirement. We adjusted for the actual labor participation rates to address this underestimation of income loss in future earnings. The current retirement age in China (typically 60 for males and 50–55 for females) is lower than many advanced economies although the government recently announced plans to gradually raise the retirement age [38].

To our knowledge this is the first study in urban China to provide comprehensive estimates of the economic burden attributable to modifiable risk factors. Previous studies have reported the attributable disease burden for a single health condition or a single risk factor [1, 39–41], with little comparison and apportioning of the attributable economic burden across multiple modifiable risk factors [42, 43]. Our findings offer vital insights into understanding the economic burden of modifiable risk factors in urban China by presenting the relative contributions of various modifiable risk factors. This can guide policymakers in prioritizing programs aimed at improving population health and mitigating economic costs.

Tobacco poses a significant threat to public health and was the single leading contributor to healthcare and societal costs. However, progress on effective tobacco control in China has generally been slow and weak [44] despite China being one of the initial signatories to the Framework Convention on Tobacco Control (WHO FCTC) in 2003 [45], and committing to achieving a 100% smoke-free policy in indoor public places and workplaces by 2011. The overall smoking prevalence in China decreased only slightly from 28.0% in 2015 to 26.7% in 2018 [46], with still alarmingly high smoking rates among males (50.5% vs 2.1% in females). This is consistent with our findings that the economic burden attributable to tobacco remained stable from 2015 to 2020 and that reducing tobacco use has the single greatest potential among the modifiable risk factors to reduce the economic burden of preventable ill-health in society. As there is currently no nationwide smoke-free legislation in China with implementation in only seven cities (including Shanghai), it is essential to widen coverage as this only covers 14% of the population. Tobacco control is a national priority with the Chinese government releasing the Healthy China 2030 Action Plan in 2019 [47] that set goals to reduce the smoking prevalence to 20% and increase the proportion of the population protected by comprehensive smoke-free regulations to 80% by 2030. Future studies should continue to monitor the impact on the economic burden when evaluating the Healthy China 2030 Action Plan.

While alcohol use ranks as the fourth leading risk factor for attributable healthcare costs, it is the second leading risk factor for productivity loss and societal cost (after tobacco). The past few decades have seen rapid economic growth in China, but also a striking increase in alcohol consumption larger than much of the world [48]. Encouragingly, recent per capita alcohol consumption decreased from 7.3 L in 2015 to 5.7 L in 2019 [49]. Notably, alcohol use was the primary modifiable risk factor for young adults aged 20–44, accounting for 19% of the total attributable societal economic burden in this group. Alcohol use leads to social harms as well as health harms; although the Healthy China 2030 Action Plan [47] recognizes the importance of reducing alcohol consumption, specific alcohol control-related policies for young adults are lacking [50].

Our estimates of the economic burden in 2020 occurred during the COVID-19 pandemic that reduced healthcare utilization in both high- and lower-income countries [51, 52]. We observed a nearly 30% reduction in hospitalizations in 2020 relative to 2015. The notable reduction in the average length of hospital stays observed in Shanghai could be attributed to several factors. Many hospitals took COVID-19 pandemic measures in 2020 such as expediting the turnover of beds, creating isolation areas, and prioritizing admissions for patients with COVID-19 which contributed to rapid decreases in length of stay. Shanghai has also been a pioneer in China by piloting the diagnosis-related group (DRG) payment system in public hospitals starting in May 2019 [53]. The use of DRGs in healthcare reimbursement has affected the delivery of care by promoting consistent evidence-based treatments that shorten hospital stays. Yet despite this decline in admissions and length of hospitalization, medical expenditure per admission increased by 78% from 2015 to 2020. The rise in average healthcare expenditure per admission is likely due to the changing management of public hospitals to control costs, reduce unnecessary services, and improve hospital standards. These measures incentivized tertiary hospitals to treat patients with complex conditions, and encouraged patients with routine needs to seek treatment outside hospitals in primary care [54], resulting in more intensive treatment and higher costs per admission in secondary and tertiary medical institutions. Further investigation of the long-term implications of the COVID-19 pandemic on healthcare utilization patterns and the attributable economic burden of risk factors are warranted. Exploring the implementation of the diagnosis-related group payment system and other cost-control measures on healthcare delivery and expenditures could help to improve efficiency and address the economic burdens.

There were several limitations to our study. First, it shares the same methodological assumptions and constraints as the GBD study. The Comparative Risk Assessment methodology commonly used for estimating the burden attributable to risk factors is limited by variable risk exposure definitions and data quality. Despite these issues, the GBD data is currently the most comprehensive estimate of health burden related to modifiable risk factors. Second, this study attributes the economic costs of modifiable risk factors using an epidemiological approach since direct PAFs to link risk factors and economic burden do not exist [55]. Thus, we used PAFs that reflect the relationship between risk factors and health outcomes to model associations between risk factors and costs. Third, as subnational GBD data for China was unavailable, we used data for mainland China, which may lead to inaccurate estimates at the urban level. Fourth, we obtained medical expenses from admissions in public and private hospitals but lack data on outpatients or emergency care, though the high cost of inpatient care typically accounts for the largest proportion of healthcare expenditure. This leads to underestimation of the healthcare costs for modifiable risk factors. Fifth, the additional economic value of unpaid labor, such as domestic work and caregiving, was unaccounted leading to underestimation of productivity losses and likely to particularly affect women [56, 57]. Specific risk factors, such as tobacco use, could also incur additional unaccounted costs such as workplace absenteeism [58]. Sixth, the attributable burden is typically based on current exposure and outcomes whereas the actual temporal relationship between risk and outcomes may vary. Therefore, we cannot directly infer cost reductions based on risk exposure reductions although we can estimate the relative contribution of risk factors to the economic costs. Seventh, our estimates were confined to healthcare costs and absenteeism losses for a single year, with foregone wages estimated from the age of death for premature mortality. Potential healthcare cost savings from premature mortality were not included in this study. Eighth, in estimating premature mortality, we assumed that the age-sex distribution of mortality in hospital reflects the general population which may introduce bias if the distributions deviate. Access to accurate population disease-age-sex-specific mortality data would lead to better estimates in future. Ninth, in 2020, there was a noticeable decrease in hospital admissions, lengths of stay, and deaths, largely due to the impact of COVID-19 on health-seeking behavior that reduced healthcare expenditure. Lastly, our study estimated the economic burden of modifiable risk factors at level 2 of the GBD study and we were unable to decompose these factors further. For instance, we could not determine the specific components that contribute to the dietary risk burden. Future research is needed to analyze these risks to enable targeted public health interventions.

## Conclusions

The economic burden of diseases from modifiable risk factors is considerable on the healthcare system and society at large. It underscores the urgent for policy-level interventions to address the major modifiable risk factors (tobacco, alcohol and dietary risks) that collectively account for half of the total attributable economic costs. Due to variations by age and sex, interventions should be tailored to target different groups, focusing on behavioral factors in younger adults aged 20–44, and a combination of behavioral and metabolic risks in those aged 45 and above. Policy initiatives aimed at reducing tobacco and alcohol consumption in males should be prioritized to reduce the economic burden associated with modifiable risk factors.

## Supplementary Information

Additional file 1: Fig S1. Attributable cost of health conditions by age and sex in 2020. Table S1. Modifiable risk factors (*n* = 20). Table S2. ICD-10 codes for 22 health conditions. Table S3. Annual income of employed residents in Shanghai in 2015 and 2020. Table S4. Annual income growth rates of employed residents in Shanghai, 2008–2021. Table S5. Labor force participation rate of residents in Shanghai, 2010–2020. Table S6. Mortality rates by age and sex of Shanghai residents in 2015 and 2020. Table S7. Consumer price indices in Shanghai, 2015–2022. Table S8. Inpatient hospital admissions by age and sex in 2015 and 2020. Table S9. Healthcare expenditure of hospitalizations in 2015 and 2020. Table S10. Attributable cost of health conditions by modifiable risk factor in 2020. Table S11. Attributable healthcare cost, productivity loss, and societal cost by modifiable risk factor in 2015 and 2020. Table S12. Mean lengths of stay of hospitalizations in Shanghai in 2015 and 2020. Table S13. Sensitivity analysis adjusting wage rates, wage growth rates, and discount rates in 2020. Table S14. Sensitivity analysis varying retirement ages in 2020. Text S1. Population attributable fraction (PAF). Text S2. Cost estimation for each health condition.

## Data Availability

The data that support the findings of this study are available from the authors but restrictions apply to the availability of these data, which were used under license from Shanghai Municipal People’s Government for the current study, and so are not publicly available.

## Abbreviations

GBD: Global Burden of Diseases Study
DALYs: Disability-adjusted life-years
PAF: Population attributable fraction
GDP: Gross domestic product
ICD-10: Classification of Diseases, 10th Revision
CHIP: Chinese Household Income Project
UI: Uncertainty interval
