# Supplementary material for: Economic costs attributable to modifiable risk factors: an analysis of 24 million urban residents in China
Source: BMC Med. 2024 Nov 21;22:549. doi: 10.1186/s12916-024-03772-7 (PMC11580671; doi:10.1186/s12916-024-03772-7)
Supplement: Supplementary file 1 — Additional file 1: Fig S1. Attributable cost of health conditions by age and sex in 2020. Table S1. Modifiable risk factors (n = 20). Table S2. ICD-10 codes for 22 health conditions. Table S3. Annual income of employed residents in Shanghai in 2015 and 2020. Table S4. Annual income growth rates of employed residents in Shanghai, 2008–2021. Table S5. Labor force participation rate of residents in Shanghai, 2010–2020. Table S6. Mortality rates by age and sex of Shanghai residents in 2015 and 2020. Table S7. Consumer price indices in Shanghai, 2015–2022. Table S8. Inpatient hospital admissions by age and sex in 2015 and 2020. Table S9. Healthcare expenditure of hospitalizations in 2015 and 2020. Table S10. Attributable cost of health conditions by modifiable risk factor in 2020. Table S11. Attributable healthcare cost, productivity loss, and societal cost by modifiable risk factor in 2015 and 2020. Table S12. Mean lengths of stay of hospitalizations in Shanghai in 2015 and 2020. Table S13. Sensitivity analysis adjusting wage rates, wage growth rates, and discount rates in 2020. Table S14. Sensitivity analysis varying retirement ages in 2020. Text S1. Population attributable fraction (PAF). Text S2. Cost estimation for each health condition. [file 12916_2024_3772_MOESM1_ESM.pdf]

Economic costs attributable to modifiable risk factors: an analysis of 24 million urban residents in China

Additional file 1

Text S1. Population attributable fraction (PAF) 2

Table S1. Modifiable risk factors (n=20) 3

Table S2. ICD-10 codes for 22 health conditions 4

Table S3. Annual income of employed residents in Shanghai in 2015 and 2020 7

Table S4. Annual income growth rates of employed residents in Shanghai, 2008-2021 8

Table S5. Labor force participation rate of residents in Shanghai, 2010-2020 9

Table S6. Mortality rates by age and sex of Shanghai residents in 2015 and 2020 10

Text S2. Cost estimation for each health condition 11

Table S7. Consumer price indices in Shanghai, 2015-2022 12

Table S8. Inpatient hospital admissions by age and sex in 2015 and 2020 13

Table S9. Healthcare expenditure of hospitalizations in 2015 and 2020 14

Table S10. Attributable cost of health conditions by modifiable risk factor in 2020 15

Table S11. Attributable healthcare cost, productivity loss, and societal cost by modifiable risk factor in 2015 and 2020 19

Figure S1. Attributable cost of health conditions by age and sex in 2020 22

Table S12. Mean lengths of stay of hospitalizations in Shanghai in 2015 and 2020 23

Table S13. Sensitivity analysis adjusting wage rates, wage growth rates, and discount rates in 2020 24

Table S14. Sensitivity analysis varying retirement ages in 2020 26

Text S1. Population attributable fraction (PAF)

$$PAF = \frac{P_{pop} \times (RR - 1)}{P_{pop} \times (RR - 1) + 1}$$

Where, P_{pop} is proportion of the population exposed to the risk factor. RR is the relative risk or risk ratio associated with the exposure. The PAF can range from 0% to 100%, where 0% indicates that the risk factor has no impact on the health condition, and 100% indicates that all cases of the health condition are attributable to the risk factor. PAF is an important measure in public health as it helps identify the potential impact of interventions targeting specific risk factors on the overall disease burden in a population.

Adjusted PAF across different exposures:

$$PAF_{1..i} = 1 - \prod_{1}^{i}(1 - PAF_i)$$

Where, PAF is the population attributable fraction, i is each individual risk factor. This approach assumes that the risk factors are independent, and the joint effects are multiplicative.

Table S1. Modifiable risk factors (n=20)

**Risk factors**	
Air pollution	Intimate partner violence
Alcohol use	Kidney dysfunction
Child and maternal malnutrition	Low bone mineral density
Childhood sexual abuse and bullying	Low physical activity
Dietary risks	Non-optimal temperature
Drug use	Occupational risks
High LDL cholesterol	Other environmental risks
High body-mass index	Tobacco
High fasting plasma glucose	Unsafe sex
High systolic blood pressure	Unsafe water, sanitation, and handwashing

Table S2. ICD-10 codes for 22 health conditions

Cause	ICD-10
Nutritional deficiencies	D50.1-D50.8, D51-D52.0, D52.8-D53.9, E00-E02, E40-E46.9, E51-E61.9, E63-E64.0, E64.2-E64.9, M12.1
Neoplasms	C00-C13.9, C15-C22.8, C23-C25.9, C30-C34.9, C37-C38.8, C40-C41.9, C43-C45.9, C47-C54.9, C56-C57.8, C60-C63.8, C64-C67.9, C68.0-C68.8, C69.0-C69.8, C70-C73.9, C75-C75.8, C81-C86.6, C88-C91.0, C91.2-C91.3, C91.6, C92-C92.6, C93-C93.1, C93.3, C93.8, C94-C96.9, D00.1-D00.2, D01.0-D01.3, D02.0-D02.3, D03-D06.9, D07.0-D07.2, D07.4-D07.5, D09.0, D09.2-D09.3, D09.8, D10.0-D10.7, D11-D12.9, D13.0-D13.7, D14.0-D14.3, D15-D16.9, D22-D24.9, D26.0-D27.9, D28.0-D28.1, D28.7, D29.0-D29.8, D30.0-D30.8, D31-D36, D36.1-D36.7, D37.1-D37.5, D38.0-D38.5, D39.1-D39.2, D39.8, D40.0-D40.8, D41.0-D41.8, D42-D43.9, D44.0-D44.8, D45-D47.9, D48.0-D48.6, D49.2-D49.4, D49.6, K62.0-K62.1, K63.5, N60-N60.9, N84.0-N84.1, N87-N87.9
Cardiovascular diseases	B33.2, G45-G46.8, I01-I01.9, I02.0, I05-I09.9, I11-I11.9, I20-I25.9, I27.0, I27.2, I28-I28.9, I30-I31.1, I31.8-I37.8, I38-I41.9, I42.1-I42.8, I43-I43.9, I47-I48.9, I51.0-I51.4, I60-I63.9, I65-I66.9, I67.0-I67.3, I67.5-I67.6, I68.0-I68.2, I69.0-I69.3, I70.2-I70.8, I71-I73.9, I77-I83.9, I86-I89.0, I89.9, I98, K75.1
Digestive diseases	B18-B18.9, I84-I85.9, I98.2, K20-K20.9, K22-K22.6, K22.8-K29.9, K31-K31.8, K35-K38.9, K40-K42.9, K44-K46.9, K50-K52, K52.2-K52.9, K55-K62, K62.2-K62.6, K62.8-K62.9, K64-K64.9, K66.8, K67, K68, K70-K70.3, K71.7, K73-K75, K75.2, K75.4-K76.2, K76.4-K77, K77.8, K80-K83.9, K85-K86.9, K90-K90.9, K92.8, K93.8, M09.1
Mental disorders	F24, F50.0-F50.5
Unintentional injuries	L55-L55.9, L56.3, L56.8-L56.9, L58-L58.9, N30.4, W00-W46.2, W49-W62.9, W64-W70.9, W73-W75.9, W77-W81.9, W83-W94.9, W97.9, W99-X06.9, X08-X39.9, X47-X48.9, X50-X54.9, X57-X58.9, Y40-Y84.9, Y88-Y88.3
Self-harm and interpersonal violence	U00-U03, X60-X64.9, X66-X83.9, X85-Y08.9, Y35-Y38.9, Y87.0-Y87.1, Y89.0-Y89.1
Respiratory infections and tuberculosis	A10-A14, A15-A19.9, A48.1, A70, B90-B90.9, B97.4-B97.6, H70-H70.9, J00-J02.8, J03-J03.8, J04-J04.2, J05-J05.1, J06.0-J06.8, J09-J15.8, J16-J16.9, J20-J21.9, J36-J36.0, J91.0, K67.3, K93.0, M49.0, N74.1, P23.0-P23.4, P37.0, U04-U04.9, U84.3
Chronic respiratory diseases	D86-D86.2, D86.9, G47.3, J30-J35.9, J37-J39.9, J41-J46.9, J60-J63.8, J65-J68.9, J70, J70.8-J70.9, J82, J84-J84.9, J91, J91.8-J92.9
Neurological disorders	F00-F02.0, F02.2-F02.3, F02.8-F03.9, G10-G13.8, G20-G20.9, G23-G24, G24.1-G25.0, G25.2-G25.3, G25.5, G25.8-G26.0, G30-G31.1, G31.8-G31.9, G35-G37.9, G40-G41.9, G61-G61.9, G70-

	G71.1, G71.3-G72, G72.2-G73.7, G90-G90.9, G95-G95.9, M33-M33.9
Other non-communicable diseases	D25-D26, D28.2, D52.1, D55-D58.9, D59.0-D59.3, D59.5-D59.6, D60-D61.9, D64.0, D66-D67, D68.0-D69.8, D70-D70.2, D70.4-D75.8, D76-D78.8, D86.8, D89-D89.2, E03-E07.1, E09-E09.9, E15.0, E16.0-E16.9, E20-E24.3, E24.8-E34, E34.1-E34.8, E36-E36.8, E65-E68, E70-E85.2, E88-E89.9, G21.0-G21.1, G24.0, G25.1, G25.4, G25.6-G25.7, G71.2, G72.0, G93.7, G97-G97.9, I95.2-I95.3, I97-I97.9, I98.9, J70.0-J70.5, J95-J95.9, K43-K43.9, K52.0, K62.7, K91-K91.9, K94-K95.8, M87.1, N10-N12.9, N13.6, N14-N15, N15.1-N16.8, N20-N23.0, N25-N28.1, N29-N30.3, N30.8-N32.0, N32.3-N32.4, N34-N34.3, N36-N36.9, N39-N39.2, N41-N41.9, N44-N44.0, N45-N45.9, N49-N49.9, N65-N65.1, N72-N72.0, N75-N77.8, N80-N81.9, N83-N83.9, N99-N99.9, P96.0, P96.2, P96.5, Q00-Q07.9, Q10.4-Q18.9, Q20-Q28.9, Q30-Q36, Q37-Q45.9, Q50-Q60.6, Q63-Q86, Q86.1-Q87.8, Q89-Q89.8, Q90-Q93.9, Q95-Q99.8, R50.2, R95-R95.9
Sense organ diseases	B30-B30.9, H00-H02.8, H02.82-H02.9, H03.0-H05.329, H05.34-H05.419, H05.8-H06.3, H10-H11.9, H13-H13.8, H15-H22.8, H25-H28.8, H30-H36.8, H40-H40.9, H42-H44.539, H44.8-H55.89, H57-H58.9, H60-H62.8, H71-H75.83, H80-H83.93, H90-H91, H91.1-H94.83, Q16-Q16.9, R43-R44.9, Z01.0-Z01.12, Z13.5, Z41.3, Z52.5, Z82.1-Z82.2, Z83.5-Z83.6, Z94.7, Z97.3-Z97.4
Transport injuries	V00-V86.9, V87.2-V87.3, V88.2-V88.3, V90-V98.8
Enteric infections	A00-A00.9, A01.0-A09.9, A80-A80.9, K52.1, R19.7
Musculoskeletal disorders	I27.1, I67.7, L93-L93.2, M00-M03.0, M03.2-M03.6, M05-M09.0, M09.2-M09.8, M30-M32.9, M34-M36.8, M40-M43.1, M65-M65.0, M71.0-M71.1, M80-M82.8, M86.3-M86.4, M87-M87.0, M88-M89.0, M89.5, M89.7-M89.9
Substance use disorders	E24.4, F10-F16.9, F18-F18.9, G31.2, G62.1, G72.1, P04.3-P04.4, P96.1, Q86.0, R78.0-R78.5, X45-X45.9, X65-X65.9, Y15-Y15.9
Diabetes and kidney diseases	D63.1, E10-E11.9, I12-I13.9, N00-N08.8, N15.0, N18-N18.9, P70.2, Q61-Q62.8
HIV/AIDS and sexually transmitted infections	A50-A58, A60-A60.9, A63-A63.8, B20-B24.9, B63, F02.4, I98.0, K67.0-K67.2, M03.1, M73.0-M73.1
Other infectious diseases	A20-A28.9, A32-A39.9, A48.2, A48.4-A48.5, A65-A65.0, A69-A69.1, A74, A74.8-A74.9, A81-A81.9, A83-A89.9, B00-B06.9, B10-B10.8, B15-B16.2, B17.0, B17.2, B19.1, B25-B27.9, B29.4, B33, B33.3-B33.8, B47-B48.8, B91, B94.1, B95-B95.5, D70.3, D89.3, F02.1, F07.1, G00.0-G00.8, G03-G03.8, G04-G05.8, G14-G14.6, G21.3, I00, I02, I02.9, I98.1, K67.8, K75.3, K76.3, K77.0, M49.1, M89.6, P35-P35.9, P37, P37.2, P37.5-P37.9, U82-U84, U85-U89, Z16-Z16.3

Maternal and neonatal disorders	C58-C58.0, N96, N98-N98.9, O00-O07.9, O09-O16.9, O20-O26.9, O28-O36.9, O40-O48.1, O60-O77.9, O80-O92.7, O96-O98.6, O98.8-P04.2, P04.5-P05.9, P07-P15.9, P19-P22.9, P24-P29.9, P36-P36.9, P38-P39.9, P50-P61.9, P70-P70.1, P70.3-P72.9, P74-P78.9, P80-P81.9, P83-P84, P90-P94.9, P96, P96.3-P96.4, P96.8

Table S3. Annual income of employed residents in Shanghai in 2015 and 2020

Annual income	Year 2015		Year 2020	
Age	Male (RMB)	Female (RMB)	Male (RMB)	Female (RMB)
20-24 years	47898	45707	83377	79562
25-29 years	75099	61258	130724	106631
30-34 years	95670	67721	166533	117882
35-39 years	98317	69761	171140	121433
40-44 years	93537	59818	162819	104125
45-49 years	81248	59217	141428	103078
50-54 years	75343	53097	131148	92426
55-59 years	66802	44533	116282	77518
60-64 years	44104	27953	76772	48658
65-69 years	31546	20428	54912	35559
70-74 years	23942	15892	41676	27663
75-79 years	6639	4506	33406	7844
80+ years	NA	NA	NA	NA

Notes: Income distribution of residents in urban China by age and sex from the Chinese Household Income Project (CHIP) survey in 2018 (http://www.ciidbnu.org/chip/) standardized by average income level of Shanghai residents of Shanghai residents from the Shanghai Statistical Yearbook (https://tjj.sh.gov.cn/tjnj/).

Standardization steps:

(1) Extract mean income level of Shanghai residents in 2015 and 2020 from the Shanghai Statistical Yearbook.

(2) Calculate the mean individual income level of residents from CHIP 2018 urban sample data.

(3) Divide the step 1 figures by the step 2 figures to get the standardized rate of mean income.

(4) Subtract the mean income level for urban residents by age and gender from individual income level in the CHIP 2018 survey urban sample data.

(5) Multiply the result from step 4 by the standardized rate from step 3 to get the standardized income by age and gender for Shanghai residents.

Table S4. Annual income growth rates of employed residents in Shanghai, 2008-2021

Year	Average income (RMB)	Annual growth rate (nominal)
2008	39502	-
2009	42789	8.3%
2010	46757	9.3%
2011	51968	11.1%
2012	56300	8.3%
2013	60435	7.3%
2014	65417	8.2%
2015	71268	8.9%
2016	78045	9.5%
2017	85528	9.6%
2018	105176	23.0%
2019	114962	9.3%
2020	124056	7.9%
2021	136757	10.2%
Mean	-	10.1%

Notes: Data source from Shanghai Statistical Yearbook (https://tjj.sh.gov.cn/tjnj/). In sensitivity analyses, we generated simulated data for the income growth rate from a lognormal distribution using actual annual salary data of Shanghai workers from 2008 to 2021 (mean value of 10% and standard deviation of 4%).

Table S5. Labor force participation rate of residents in Shanghai, 2010-2020

Age	Year 2010		Year 2015		Year 2020	
	Male	Female	Male	Female	Male	Female
20-24 years	80%	72%	80%	72%	81%	73%
25-29 years	96%	84%	95%	86%	95%	88%
30-34 years	97%	84%	97%	87%	98%	89%
35-39 years	97%	85%	98%	89%	98%	92%
40-44 years	96%	84%	97%	89%	98%	93%
45-49 years	92%	71%	95%	80%	98%	89%
50-54 years	83%	26%	90%	50%	96%	73%
55-59 years	64%	13%	79%	30%	94%	47%
60-64 years	16%	7%	37%	14%	58%	21%
65-69 years	9%	4%	22%	9%	35%	13%
70-74 years	3%	2%	11%	4%	18%	7%
75-79 years	1%	0%	4%	2%	7%	3%

Notes: Data source from Shanghai Population Census Yearbook 2010 and Shanghai Population Census Yearbook 2020 (https://tjj.sh.gov.cn/tjnj_rkpc/index.html). We assumed the labor force participation rates had a linear trend between 2015 to 2020. We inferred the 2015 employment rate by age and sex using linear interpolation of the employment data by age and sex obtained from the 2010 and 2020 census.

Table S6. Mortality rates by age and sex of Shanghai residents in 2015 and 2020

Year 2015	All deaths			Mortality rate (‰)			Death in hospitals		
**Age**	**Male**	**Female**	**Total**	**Male**	**Female**	**Overall**	**Male**	**Female**	**Total**
20-24 years	185	82	267	0.15	0.07	0.12	9	9	18
25-29 years	212	110	322	0.17	0.09	0.13	25	30	55
30-34 years	282	154	436	0.23	0.14	0.19	47	47	94
35-39 years	440	246	686	0.40	0.25	0.33	72	62	134
40-44 years	654	331	984	0.67	0.38	0.53	109	72	181
45-49 years	1,191	609	1,800	1.26	0.72	1.00	216	128	344
50-54 years	2,169	1,053	3,222	2.31	1.21	1.78	623	296	919
55-59 years	3,489	1,549	5,037	4.04	1.86	2.97	1,327	635	1,962
60-64 years	5,022	2,164	7,185	7.02	3.16	5.11	1,731	788	2,519
65-69 years	6,079	2,850	8,929	11.38	5.61	8.50	1,694	809	2,503
70-74 years	7,294	3,899	11,193	20.57	10.82	15.58	1,492	797	2,289
75-79 years	9,301	6,598	15,899	37.18	22.55	29.34	1,936	1,324	3,260
80+ years	29,099	35,862	64,961	104.34	85.71	93.15	6,792	7,078	13,870
Total	65,414	55,504	120,918	6.13	5.52	5.84	16,073	12,075	28,148
**Year 2020**									
20-24 years	79	36	115	0.09	0.05	0.07	5	3	8
25-29 years	108	63	171	0.09	0.06	0.08	13	8	21
30-34 years	237	129	366	0.17	0.10	0.14	16	18	34
35-39 years	409	234	643	0.35	0.22	0.29	38	25	63
40-44 years	538	279	817	0.55	0.32	0.44	48	34	82
45-49 years	777	415	1,192	0.82	0.48	0.66	64	35	99
50-54 years	1,261	650	1,911	1.36	0.78	1.09	114	53	167
55-59 years	3,007	1,205	4,212	3.40	1.43	2.44	189	76	265
60-64 years	5,857	2,381	8,238	6.60	2.63	4.60	393	175	568
65-69 years	8,059	3,544	11,603	10.23	4.38	7.27	470	239	709
70-74 years	8,600	4,102	12,702	17.85	8.44	13.12	478	228	706
75-79 years	8,133	4,856	12,989	31.71	17.58	24.38	337	200	537
80+ years	33,138	40,514	73,652	98.28	81.33	88.17	899	770	1,669
Total	70,203	58,408	128,611	6.28	5.57	5.93	3,064	1,864	4,928

Notes: Data source from Shanghai Population Census Yearbook 2010 and Shanghai Population Census Yearbook 2020 (https://tjj.sh.gov.cn/tjnj_rkpc/index.html). We assumed the mortality rates had a linear trend between 2015 to 2020. We inferred the 2015 mortality rate by age and sex using linear interpolation of the mortality data by age and sex obtained from the 2010 and 2020 census. To estimate the number of premature deaths in Shanghai, we adjusted the age-sex specific hospital premature death counts by the age-sex specific mortality rates of residents in Shanghai.

Text S2. Cost estimation for each health condition

- Attributable cost = Population attributable fraction (PAF) × Societal cost
- Societal cost = Healthcare cost + Productivity loss
- Healthcare cost = $\sum_i$ hospitalization charge × cost to charge ratio;
- Productivity loss =loss due to absenteeism + loss due to premature death
- Loss due to absenteeism $= \sum_s \sum_a$ (daily income × labor force participation rate × length of stay)
- Daily $\text{wages}_{s,a} = \text{annual income}_{s,a}/250$ workdays per year
- Loss due to premature death $= \sum_s \sum_a$ (future earning × labor force participation rate × no. of deaths)
- Future $\text{earning}_{s,a} = \text{annual wages}_{s,a} + \text{annual wages}_{s,a} \times (1 + \text{income growth rate} - \text{discount rate})^j$
- Number of $\text{deaths}_{s,a} =$ no. of deaths in hospital $_{s,a} \times$ death $\text{adjustment}_{s,a}$
- Death $\text{adjustment}_{s,a} =$ no. of general $\text{death}_{s,a} / \sum_d$ no. of deaths in hospital $_{s,a}$

Data source of components used for cost estimation:

Estimation components	Data source
Population attributable fraction (PAF)	GBD study
Healthcare cost:<br>- Hospitalization expenditure	Shanghai Municipal Health Commission covering all inpatients in hospitals in 2015 and 2020.
Productivity loss due to absenteeism:<br>- length of stay<br>- annual income<br>- labor force participation rate	CHIP 2018 survey<br>Shanghai Statistical Yearbook 2008-2021<br>Shanghai Population Census Yearbook 2020<br>(https://tjj.sh.gov.cn/tjnj/index.html)
Productivity loss due to premature death<br>- deaths<br>- future earnings<br>--- annual income,<br>--- growth rate,<br>--- discount rate	Shanghai Population Census Yearbook 2020<br>Shanghai Statistical Yearbook 2008- 2021<br>(https://tjj.sh.gov.cn/tjnj/index.html)

Notes:

- 'i' represents the index of each admission record.
- 's' represents the category index of sex groups.
- 'a' represents the category index of age groups.
- 'd' represents the category index of health conditions.
- 'j' represents the number of years from the current age until life expectancy. It ranges from 0 to the difference between life expectancy and age at death. This variable signifies the time period over which the income growth rate is compounded to calculate potential future earnings for each year.

Table S7. Consumer price indices in Shanghai, 2015-2022

Year	Medical consumer price index (preceding year=100)	General consumer price index (preceding year=100)
2015	99.3	102.4
2016	109.0	103.2
2017	106.6	101.7
2018	102.4	101.6
2019	103.3	102.5
2020	101.2	101.7
2021	98.9	101.2

Notes: Data source from Shanghai Statistical Yearbook (https://tjj.sh.gov.cn/tjnj/).

Table S8. Inpatient hospital admissions by age and sex in 2015 and 2020

Admissions	Year 2015				Year 2020			
**Age**	**Male**	**Female**	**Total**		**Male**	**Female**	**Total**	
20-24 years	5739	38871	44610	3%	5939	11725	17664	2%
25-29 years	12594	96621	109215	8%	10184	43035	53219	6%
30-34 years	16048	77936	93984	7%	16161	65352	81513	10%
35-39 years	15903	42194	58097	4%	17320	44386	61706	7%
40-44 years	16660	26914	43574	3%	18588	33684	52272	6%
45-49 years	20918	26704	47622	4%	22664	33559	56223	7%
50-54 years	37148	38354	75502	6%	30132	36155	66287	8%
55-59 years	61324	54065	115389	9%	43154	40706	83860	10%
60-64 years	76466	63894	140360	11%	53417	44623	98040	12%
65-69 years	68210	56764	124974	9%	58383	47581	105964	13%
70-74 years	55649	46346	101995	8%	41864	32230	74094	9%
75-79 years	53841	52783	106624	8%	23352	17610	40962	5%
80+ years	121968	143273	265241	20%	25116	20562	45678	5%
Total	562468	764719	1327187	100%	366274	471208	837482	100%

Notes: Inpatient hospital admissions of Shanghai residents with primary diagnoses for one of the 22 health conditions listed in Appendix 3.

Table S9. Healthcare expenditure of hospitalizations in 2015 and 2020

	Year 2015			Year 2020		
**Age**	**Male (RMB, million)**	**Female (RMB, million)**	**Total (RMB, million)**	**Male (RMB, million)**	**Female (RMB, million)**	**Total (RMB, million)**
20-44 years	797	2128	2925	1592	2891	4483
45-64 years	3300	2605	5904	4692	3833	8526
65 and above	5316	4636	9952	4937	3275	8212
Total	9413	9368	18781	11221	9999	21220

Notes: Hospitalizations of Shanghai residents with primary diagnoses for one of the 22 health conditions listed in Appendix 3. Expenditure reported are for the current year.

Table S10. Attributable cost of health conditions by modifiable risk factor in 2020

Health condition	Risk factor	Healthcare cost		Productivity loss		Societal cost	
		US$ million	% [UI]	US$ million	% [UI]	US$ million	% [UI]
Cardiovascular diseases	Air pollution	104	12.3% [9-16]	239	12.5% [9.2-16.3]	342	12.5% [9.1-16.2]
	Alcohol use	16	2.0% [0.8-4.3]	51	2.7% [1.1-5.1]	67	2.4% [1-4.8]
	Dietary risks	155	18.4% [13.4-24]	369	19.4% [15.1-23.7]	524	19.1% [14.5-23.8]
	High LDL cholesterol	74	8.8% [5.3-12.9]	212	11.1% [7.9-14.6]	286	10.4% [7.1-14]
	High body-mass index	61	7.2% [2.8-13.4]	199	10.5% [5.3-16.5]	260	9.5% [4.5-15.6]
	High fasting plasma glucose	54	6.4% [2.9-10.7]	77	4.0% [1.5-7.2]	130	4.7% [2-8.3]
	High systolic blood pressure	196	23.3% [17.8-29.6]	367	19.3% [13.9-24.3]	563	20.5% [15.1-25.9]
	Kidney dysfunction	29	3.5% [1.6-6.5]	47	2.4% [1-4.7]	76	2.8% [1.1-5.3]
	Low physical activity	6	0.7% [0.1-2.7]	5	0.2% [0-1.4]	11	0.4% [0-1.8]
	Non-optimal temperature	28	3.3% [1.6-5.6]	56	2.9% [1.3-5.1]	84	3.1% [1.4-5.3]
	Other environmental risks	21	2.5% [0.9-5.1]	26	1.4% [0.3-3]	48	1.7% [0.5-3.7]
	Tobacco	98	11.6% [8.4-14.9]	259	13.6% [10.3-17.2]	356	13.0% [9.7-16.5]
Chronic respiratory diseases	Air pollution	14	27.1% [19-35.4]	9	25.5% [18.5-32.9]	23	26.4% [18.8-34.4]
	High body-mass index	1	2.5% [0.3-7]	0	1.2% [0.1-3.7]	2	2.0% [0.2-5.6]
	Non-optimal temperature	4	8.6% [4.5-13.9]	3	8.6% [4.9-13.6]	8	8.6% [4.7-13.8]
	Occupational risks	11	21.5% [12.5-31.7]	8	20.5% [12.1-29.5]	19	21.1% [12.3-30.8]
	Tobacco	21	40.3% [31.4-49.1]	16	44.2% [36.6-51.6]	37	41.9% [33.6-50.1]
Diabetes and kidney diseases	Air pollution	6	6.3% [3.2-10]	8	3.6% [1.7-5.7]	14	4.4% [2.1-7]
	Alcohol use	1	0.6% [0-2.5]	4	1.8% [0-12.1]	5	1.4% [0-9.1]
	Dietary risks	10	9.7% [6-13.9]	13	5.6% [3-8.6]	22	6.9% [3.9-10.3]
	High body-mass index	13	13.3% [7.6-20.2]	30	13.3% [5.4-22.3]	43	13.3% [6.1-21.6]
	High fasting plasma glucose	33	33.8% [27-39.7]	78	34.7% [26-42.8]	111	34.5% [26.3-41.8]
	High systolic blood pressure	9	9.3% [5.8-13.1]	16	7.0% [2.8-11.1]	25	7.7% [3.7-11.7]
	Kidney dysfunction	16	16.4% [11.9-21.2]	61	27.4% [21.4-35.2]	77	24.0% [18.5-30.9]
	Low physical activity	1	1.1% [0.1-4.2]	1	0.3% [0-1.2]	2	0.5% [0-2.1]

	Non-optimal temperature	2	1.9% [0.4-4.3]	5	2.1% [0.3-4.6]	7	2.0% [0.3-4.5]
	Other environmental risks	1	0.8% [0.1-2.3]	0	0.2% [0-0.7]	1	0.4% [0-1.2]
	Tobacco	7	6.7% [3.5-11.1]	9	4.0% [2.2-6.3]	16	4.9% [2.6-7.8]
Digestive diseases	Alcohol use	115	52.6% [34.2-70.6]	285	62.4% [43.7-75.7]	400	59.2% [40.6-74]
	Drug use	52	23.9% [12.2-37.5]	108	23.7% [13.3-35.9]	161	23.8% [12.9-36.4]
	High body-mass index	34	15.6% [2.6-32.3]	34	7.5% [0.3-16.1]	68	10.1% [1-21.3]
	Tobacco	17	7.9% [3.1-15.6]	30	6.5% [2.3-12.5]	47	6.9% [2.5-13.5]
Enteric infections	Air pollution	0	0.0% [0-0]	0	0.0% [0-0]	0	0.0% [0-0]
	Child and maternal malnutrition	0	0.0% [0-0]	0	0.0% [0-0]	0	0.0% [0-0]
	Unsafe water, sanitation, and handwashing	1	100.0% [100-100]	0	100.0% [100-100]	1	100.0% [100-100]
HIV/AIDS and sexually transmitted infections	Drug use	0	25.7% [13.8-38.9]	0	29.9% [16.3-44.1]	0	26.9% [14.5-40.4]
	Intimate partner violence	0	2.6% [0.4-6.2]	0	1.9% [0.3-4.4]	0	2.4% [0.4-5.7]
	Unsafe sex	1	71.6% [60.3-79.9]	0	68.3% [54.8-78.3]	1	70.7% [58.8-79.4]
Maternal and neonatal disorders	Air pollution	0	0.0% [0-0]	0	0.0% [0-0]	0	0.0% [0-0]
	Child and maternal malnutrition	39	100.0% [100-100]	20	100.0% [100-100]	59	100.0% [100-100]
Mental disorders	Childhood sexual abuse and bullying	0	52.6% [1.6-93.6]	0	53.6% [7.7-98]	0	52.7% [1.8-93.8]
	Intimate partner violence	0	36.5% [0.4-68.4]	0	25.7% [0.6-43.9]	0	36.1% [0.4-67.5]
	Other environmental risks	0	10.9% [0.1-36.2]	0	20.7% [1.3-56.2]	0	11.3% [0.2-37]
Musculoskeletal disorders	High body-mass index	12	27.1% [5.3-55.8]	8	12.1% [1.1-35.7]	20	18.1% [2.8-43.7]
	Kidney dysfunction	0	1.0% [0-4]	0	0.1% [0-0.6]	1	0.5% [0-2]
	Occupational risks	21	48.8% [17.7-74.1]	52	78.5% [42.1-91]	73	66.7% [32.3-84.3]
	Tobacco	10	23.1% [9.2-41.7]	6	9.2% [2.7-19.6]	16	14.7% [5.3-28.4]
Neoplasms	Air pollution	129	11.5% [5.2-19.3]	226	8.7% [3.8-14.6]	355	9.5% [4.2-16]

	Alcohol use	82	7.4% [3.4-13.2]	444	17.0% [10-26.1]	526	14.1% [8-22.2]
	Dietary risks	122	10.9% [4.5-19.8]	303	11.6% [5.2-19.2]	425	11.4% [5-19.4]
	Drug use	18	1.6% [0.1-4.7]	31	1.2% [0.1-4.4]	50	1.3% [0.1-4.5]
	High body-mass index	82	7.3% [0.9-17.9]	285	10.9% [2.9-24.3]	366	9.8% [2.3-22.4]
	High fasting plasma glucose	59	5.3% [0.5-17]	70	2.7% [0.1-11.3]	129	3.5% [0.2-13]
	Low physical activity	4	0.4% [0-1.8]	2	0.1% [0-0.4]	6	0.2% [0-0.9]
	Occupational risks	44	3.9% [0.9-9.1]	111	4.3% [1.1-9.6]	155	4.2% [1-9.4]
	Other environmental risks	25	2.3% [0-9.2]	48	1.8% [0-7.4]	73	2.0% [0-7.9]
	Tobacco	447	39.9% [27.5-49.7]	937	36.0% [25.3-45]	1,384	37.2% [25.9-46.4]
	Unsafe sex	108	9.6% [5.5-14.5]	147	5.7% [3.6-7.8]	255	6.9% [4.2-9.8]
Neurological disorders	Alcohol use	12	37.1% [26.4-55.6]	46	78.8% [72.4-87.4]	58	63.5% [55.5-75.7]
	High body-mass index	7	20.7% [0.1-62.2]	3	5.7% [0-25.9]	10	11.2% [0-39.2]
	High fasting plasma glucose	4	11.3% [0-44.8]	1	1.6% [0-9.3]	5	5.2% [0-22.3]
	Tobacco	10	31.0% [4.5-61]	8	13.9% [1.2-28.2]	18	20.1% [2.4-40.2]
Nutritional deficiencies	Child and maternal malnutrition	0	100.0% [100-100]	0	100.0% [100-100]	0	100.0% [100-100]
Other infectious diseases	Air pollution	0	0.0% [0-0]	0	0.0% [0-0]	0	0.0% [0-0]
	Child and maternal malnutrition	0	0.0% [0-0]	0	0.0% [0-0]	0	0.0% [0-0]
	Drug use	6	100.0% [100-100]	20	100.0% [100-100]	26	100.0% [100-100]
Respiratory infections and tuberculosis	Air pollution	1	21.9% [12.6-32.2]	0	18.4% [10.4-28.4]	1	21.0% [12-31.2]
	Alcohol use	1	19.3% [10.6-29.7]	0	21.9% [13.9-30.3]	1	20.0% [11.4-29.8]
	Child and maternal malnutrition	0	0.0% [0-0]	0	0.0% [0-0]	0	0.0% [0-0]
	High fasting plasma glucose	0	4.7% [1-10.4]	0	4.6% [0.6-11.5]	0	4.7% [0.9-10.7]
	Non-optimal temperature	1	11.0% [5.1-19.1]	0	8.1% [3.9-15.6]	1	10.3% [4.8-18.2]
	Tobacco	2	39.9% [29-50.3]	1	44.5% [34.4-54.3]	3	41.1% [30.4-51.3]

	Unsafe water, sanitation, and handwashing	0	3.2% [0.4-9]	0	2.4% [0.2-6.2]	0	3.0% [0.3-8.3]
Sense organ diseases	Air pollution	10	12.7% [3.5-25]	0	4.4% [0.3-12.9]	10	12.3% [3.3-24.4]
	High body-mass index	1	1.9% [0-8.9]	0	1.2% [0-8.1]	2	1.8% [0-8.9]
	High fasting plasma glucose	3	3.6% [0-14.6]	0	0.8% [0-5.2]	3	3.4% [0-14.2]
	Occupational risks	60	76.8% [38.3-92.9]	4	91.0% [46.2-103.8]	64	77.5% [38.7-93.5]
	Tobacco	4	5.1% [1.3-12.9]	0	2.6% [0.3-8.1]	4	4.9% [1.2-12.7]
Substance use disorders	Alcohol use	0	61.4% [43.7-78.7]	0	63.1% [45-81]	0	61.7% [43.9-79.1]
	Childhood sexual abuse and bullying	0	3.4% [0-13.9]	0	4.2% [0-14.5]	0	3.5% [0-14]
	Drug use	0	35.2% [20.5-49.1]	0	32.7% [20.3-45.1]	0	34.8% [20.5-48.5]
Unintentional injuries	Alcohol use	0	17.3% [5.2-39.7]	0	14.1% [4.3-33.5]	0	17.0% [5.1-39]
	Low bone mineral density	0	44.8% [31.3-55.9]	0	42.5% [27.6-57.2]	0	44.6% [30.9-56.1]
	Non-optimal temperature	0	0.0% [0-0]	0	0.0% [0-0]	0	0.0% [0-0]
	Occupational risks	0	31.2% [16-46.8]	0	39.9% [23.7-55.9]	0	32.1% [16.8-47.7]
	Tobacco	0	6.6% [1.7-15.9]	0	3.4% [0.9-8.2]	0	6.3% [1.7-15.1]

US$, US dollar 2020 prices. UI, uncertainty interval.

Table S11. Attributable healthcare cost, productivity loss, and societal cost by modifiable risk factor in 2015 and 2020

Risk factors	Healthcare cost		Productivity loss		Societal cost	
Year 2015	US$ million [UI]	% [UI]	US$ million [UI]	% [UI]	US$ million [UI]	% [UI]
Air pollution	267<br>[159-396]	11.0%<br>[6.6 - 16.4]	294<br>[149-484]	8.3%<br>[4.2 - 13.7]	560<br>[308-880]	9.4%<br>[5.2 - 14.8]
Alcohol use	199<br>[101-332]	8.3%<br>[4.2 - 13.8]	591<br>[312-888]	16.8%<br>[8.9 - 25.2]	791<br>[413-1,220]	13.3%<br>[7 - 20.6]
Child and maternal malnutrition	189<br>[189-189]	7.8%<br>[7.8 - 7.8]	45<br>[45-45]	1.3%<br>[1.3 - 1.3]	234<br>[234-234]	3.9%<br>[3.9 - 3.9]
Dietary risks	240<br>[137-369]	10.0%<br>[5.7 - 15.3]	394<br>[191-665]	11.2%<br>[5.4 - 18.9]	635<br>[328-1,034]	10.7%<br>[5.5 - 17.4]
Drug use	80<br>[37-147]	3.3%<br>[1.5 - 6.1]	161<br>[87-316]	4.6%<br>[2.5 - 9]	241<br>[123-463]	4.1%<br>[2.1 - 7.8]
High LDL cholesterol	87<br>[47-136]	3.6%<br>[1.9 - 5.6]	76<br>[53-101]	2.2%<br>[1.5 - 2.9]	163<br>[100-237]	2.7%<br>[1.7 - 4]
High body-mass index	156<br>[27-401]	6.4%<br>[1.1 - 16.6]	336<br>[56-839]	9.5%<br>[1.6 - 23.8]	492<br>[83-1,240]	8.3%<br>[1.4 - 20.9]
High fasting plasma glucose	167<br>[69-351]	6.9%<br>[2.9 - 14.5]	110<br>[30-361]	3.1%<br>[0.8 - 10.2]	277<br>[99-712]	4.7%<br>[1.7 - 12]
High systolic blood pressure	252<br>[178-323]	10.4%<br>[7.4 - 13.4]	132<br>[84-177]	3.7%<br>[2.4 - 5]	383<br>[262-500]	6.5%<br>[4.4 - 8.4]
Kidney dysfunction	63<br>[33-103]	2.6%<br>[1.4 - 4.3]	35<br>[16-61]	1.0%<br>[0.5 - 1.7]	97<br>[49-164]	1.6%<br>[0.8 - 2.8]
Low physical activity	17<br>[1-76]	0.7%<br>[0 - 3.2]	5<br>[0-44]	0.1%<br>[0 - 1.2]	22<br>[1-120]	0.4%<br>[0 - 2]
Non-optimal temperature	54<br>[26-93]	2.2%<br>[1.1 - 3.9]	31<br>[10-64]	0.9%<br>[0.3 - 1.8]	86<br>[36-157]	1.4%<br>[0.6 - 2.6]

Occupational risks	116<br>[35-193]	4.8%<br>[1.5 - 8]	143<br>[32-331]	4.1%<br>[0.9 - 9.4]	259<br>[67-524]	4.4%<br>[1.1 - 8.8]
Other environmental risks	36<br>[8-109]	1.5%<br>[0.3 - 4.5]	48<br>[2-271]	1.4%<br>[0.1 - 7.7]	84<br>[10-381]	1.4%<br>[0.2 - 6.4]
Tobacco	442<br>[291-611]	18.3%<br>[12.1 - 25.3]	772<br>[532-1,022]	21.9%<br>[15.1 - 29]	1,213<br>[823-1,633]	20.4%<br>[13.9 - 27.5]
Unsafe sex	47<br>[28-68]	1.9%<br>[1.2 - 2.8]	348<br>[279-409]	9.9%<br>[7.9 - 11.6]	394<br>[307-476]	6.6%<br>[5.2 - 8]
Others	3<br>[2-5]	0.1%<br>[0.1 - 0.2]	3<br>[2-6]	0.1%<br>[0.1 - 0.2]	5<br>[3-11]	0.1%<br>[0.1 - 0.2]
**Year 2020**						
Air pollution	264<br>[150-400]	10.4%<br>[5.9 - 15.7]	483<br>[284-718]	8.9%<br>[5.3 - 13.3]	747<br>[435-1,118]	9.4%<br>[5.5 - 14.1]
Alcohol use	228<br>[129-361]	9.0%<br>[5.1 - 14.2]	830<br>[522-1,199]	15.4%<br>[9.7 - 22.2]	1,058<br>[651-1,560]	13.3%<br>[8.2 - 19.7]
Child and maternal malnutrition	40<br>[40-40]	1.6%<br>[1.6 - 1.6]	20<br>[20-20]	0.4%<br>[0.4 - 0.4]	59<br>[59-59]	0.7%<br>[0.7 - 0.7]
Dietary risks	287<br>[169-438]	11.3%<br>[6.7 - 17.2]	685<br>[429-971]	12.7%<br>[8 - 18]	971<br>[599-1,409]	12.2%<br>[7.5 - 17.7]
Drug use	77<br>[34-142]	3.0%<br>[1.3 - 5.6]	160<br>[82-298]	3.0%<br>[1.5 - 5.5]	237<br>[116-439]	3.0%<br>[1.5 - 5.5]
High LDL cholesterol	74<br>[44-108]	2.9%<br>[1.7 - 4.3]	212<br>[151-278]	3.9%<br>[2.8 - 5.1]	286<br>[195-386]	3.6%<br>[2.5 - 4.9]
High body-mass index	211<br>[49-460]	8.3%<br>[1.9 - 18.1]	560<br>[190-1,112]	10.4%<br>[3.5 - 20.6]	771<br>[239-1,572]	9.7%<br>[3 - 19.8]
High fasting plasma glucose	153<br>[57-346]	6.0%<br>[2.2 - 13.6]	225<br>[89-534]	4.2%<br>[1.6 - 9.9]	378<br>[146-881]	4.8%<br>[1.8 - 11.1]

High systolic blood pressure	205 [155-262]	8.1% [6.1 - 10.3]	383 [271-488]	7.1% [5 - 9]	588 [427-750]	7.4% [5.4 - 9.4]
Kidney dysfunction	46 [25-78]	1.8% [1 - 3.1]	108 [66-169]	2.0% [1.2 - 3.1]	154 [91-247]	1.9% [1.1 - 3.1]
Low physical activity	11 [1-48]	0.5% [0 - 1.9]	7 [0-42]	0.1% [0 - 0.8]	19 [1-89]	0.2% [0 - 1.1]
Non-optimal temperature	35 [16-60]	1.4% [0.6 - 2.4]	64 [27-113]	1.2% [0.5 - 2.1]	99 [43-173]	1.2% [0.5 - 2.2]
Occupational risks	137 [54-224]	5.4% [2.1 - 8.8]	174 [63-325]	3.2% [1.2 - 6]	311 [117-548]	3.9% [1.5 - 6.9]
Other environmental risks	48 [8-148]	1.9% [0.3 - 5.8]	75 [6-252]	1.4% [0.1 - 4.7]	122 [14-401]	1.5% [0.2 - 5]
Tobacco	616 [413-805]	24.2% [16.3 - 31.7]	1,265 [886-1,620]	23.4% [16.4 - 30]	1,881 [1,299-2,425]	23.7% [16.4 - 30.5]
Unsafe sex	109 [63-163]	4.3% [2.5 - 6.4]	147 [94-203]	2.7% [1.7 - 3.8]	256 [157-366]	3.2% [2 - 4.6]
Others	1 [1-1]	0.0% [0 - 0.1]	0 [0-0]	0.0% [0 - 0]	1 [1-2]	0.0% [0 - 0]

US$, US dollar 2020 prices. UI, uncertainty interval.

Notes: Other risks include childhood sexual abuse and bullying, intimate partner violence, low bone mineral density, unsafe water, sanitation and handwashing.

Figure S1. Attributable cost of health conditions by age and sex in 2020

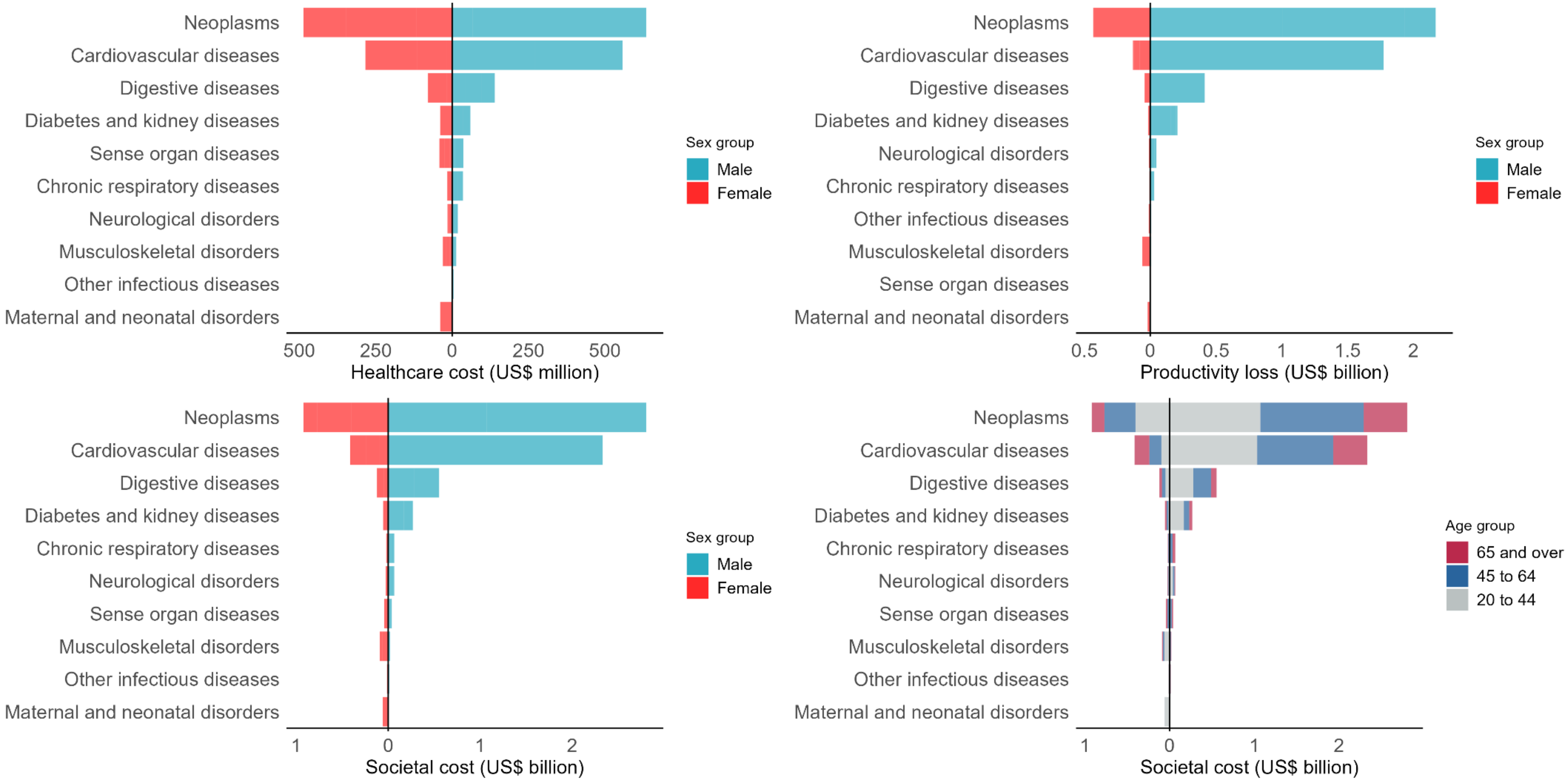

US$, US dollar 2020 prices.

Table S12. Mean lengths of stay of hospitalizations in Shanghai in 2015 and 2020

	Year 2015			Year 2020		
Age	Male (days)	Female (days)	Total (days)	Male (days)	Female (days)	Total (days)
20-24 years	7.5	5.1	5.4	6.3	4.7	5.2
25-29 years	7.4	5.2	5.5	6.5	4.6	5.0
30-34 years	7.4	5.3	5.7	5.8	4.8	5.0
35-39 years	7.6	5.7	6.2	6.0	4.9	5.2
40-44 years	8.2	6.4	7.1	6.1	5.3	5.6
45-49 years	9.1	7.4	8.1	6.6	5.9	6.2
50-54 years	10.0	8.0	9.0	7.1	6.3	6.7
55-59 years	11.1	8.6	9.9	7.1	6.4	6.8
60-64 years	10.8	9.0	10.0	7.2	6.5	6.9
65-69 years	11.6	10.0	10.9	7.5	6.8	7.2
70-74 years	13.0	12.1	12.6	7.8	7.2	7.6
75-79 years	15.9	16.3	16.1	8.6	7.6	8.1
80+ years	23.8	28.7	26.5	13.3	10.4	12.0
Total	14.0	12.0	12.8	7.6	6.0	6.7

Notes: Hospitalizations of Shanghai residents with primary diagnoses for one of the 22 health conditions listed in Appendix 3.

Table S13. Sensitivity analysis adjusting wage rates, wage growth rates, and discount rates in 2020

	Healthcare cost	Productivity loss	Societal cost
**Scenario 1 Salary**	**US$ million [UI]**	**US$ million [UI]**	**US$ million [UI]**
All conditions	2,541 [1,408-4,083]	12,038 [7,093-18,605]	14,578 [8,501-22,688]
Cardiovascular diseases	842 [542-1,227]	4,249 [2,836-5,913]	5,091 [3,378-7,140]
Chronic respiratory diseases	51 [35-70]	82 [59-108]	134 [94-178]
Diabetes and kidney diseases	99 [65-141]	499 [314-752]	598 [378-893]
Digestive diseases	219 [114-341]	1,020 [607-1,430]	1,239 [721-1,771]
Maternal and neonatal disorders	39 [39-39]	44 [44-44]	83 [83-83]
Musculoskeletal disorders	44 [14-77]	147 [68-217]	191 [82-294]
Neoplasms	1,121 [544-1,974]	5,808 [3,018-9,882]	6,929 [3,562-11,857]
Neurological disorders	34 [10-75]	129 [95-195]	163 [106-270]
Sense organ diseases	79 [34-121]	9 [4-13]	88 [38-134]
Others	13 [11-16]	50 [48-52]	63 [59-68]
**Scenario 2 Income growth 5%**			
All conditions	2,541 [1,408-4,083]	6,607 [3,883-10,216]	9,148 [5,292-14,299]
Cardiovascular diseases	842 [542-1,227]	2,296 [1,527-3,220]	3,138 [2,068-4,447]
Chronic respiratory diseases	51 [35-70]	63 [45-83]	114 [80-153]
Diabetes and kidney diseases	99 [65-141]	216 [138-317]	315 [203-458]
Digestive diseases	219 [114-341]	547 [326-770]	766 [441-1,111]
Maternal and neonatal disorders	39 [39-39]	27 [27-27]	67 [67-67]
Musculoskeletal disorders	44 [14-77]	77 [33-116]	121 [47-193]
Neoplasms	1,121 [544-1,974]	3,282 [1,718-5,532]	4,403 [2,263-7,507]
Neurological disorders	34 [10-75]	61 [37-108]	95 [47-184]
Sense organ diseases	79 [34-121]	9 [4-13]	88 [38-134]
Others	13 [11-16]	29 [28-31]	42 [38-47]
**Scenario 3 Undiscounted**			
All conditions	2,541 [1,408-4,083]	8,165 [4,804-12,620]	10,706 [6,212-16,703]
Cardiovascular diseases	842 [542-1,227]	2,862 [1,905-4,002]	3,704 [2,447-5,229]

Chronic respiratory diseases	51<br>[35-70]	70<br>[50-91]	121<br>[85-162]
Diabetes and kidney diseases	99<br>[65-141]	287<br>[182-425]	386<br>[247-566]
Digestive diseases	219<br>[114-341]	683<br>[407-959]	902<br>[521-1,300]
Maternal and neonatal disorders	39<br>[39-39]	32<br>[32-32]	71<br>[71-71]
Musculoskeletal disorders	44<br>[14-77]	97<br>[43-145]	141<br>[57-222]
Neoplasms	1,121<br>[544-1,974]	4,011<br>[2,095-6,783]	5,132<br>[2,639-8,757]
Neurological disorders	34<br>[10-75]	79<br>[52-133]	113<br>[62-208]
Sense organ diseases	79<br>[34-121]	9<br>[4-13]	88<br>[38-134]
Others	13<br>[11-16]	35<br>[34-37]	49<br>[44-53]
	**Healthcare cost**	**Productivity loss**	**Societal cost**
**Scenario 4**<br>**Discount rate 5%**	**US$ million [UI]**	**US$ million [UI]**	**US$ million [UI]**
All conditions	2,541<br>[1,408-4,083]	5,079<br>[2,982-7,862]	7,620<br>[4,390-11,945]
Cardiovascular diseases	842<br>[542-1,227]	1,739<br>[1,154-2,447]	2,581<br>[1,696-3,674]
Chronic respiratory diseases	51<br>[35-70]	55<br>[40-72]	106<br>[74-142]
Diabetes and kidney diseases	99<br>[65-141]	156<br>[100-227]	255<br>[165-367]
Digestive diseases	219<br>[114-341]	415<br>[248-586]	634<br>[362-927]
Maternal and neonatal disorders	39<br>[39-39]	23<br>[23-23]	62<br>[62-62]
Musculoskeletal disorders	44<br>[14-77]	57<br>[23-87]	101<br>[37-164]
Neoplasms	1,121<br>[544-1,974]	2,558<br>[1,344-4,299]	3,679<br>[1,888-6,273]
Neurological disorders	34<br>[10-75]	45<br>[24-85]	78<br>[34-160]
Sense organ diseases	79<br>[34-121]	9<br>[4-13]	88<br>[38-134]
Others	13<br>[11-16]	23<br>[22-25]	36<br>[32-41]

US$, US dollar 2020 prices. UI, uncertainty interval.

Notes: Other conditions include mental disorders, nutritional deficiencies, other non-communicable diseases, self-harm and interpersonal violence, substance use disorders, unintentional injuries, enteric infections, HIV/AIDS and sexually transmitted infections, respiratory infections and tuberculosis, and other infectious diseases.

Attributable cost in the year 2020. Scenario 1: Raise salary in Shanghai in 2020 (base value) to an advanced country. Here we use Singapore's salary income as the reference level. Scenario 2: Reduce income growth rate from 10% (base value) to 5% based on Scenario 1. Scenario 3: Change annual discount rate from 3% (base value) to 0% based on Scenario 2. Scenario 4: Change annual discount rate from 3% (base value) to 5% based on Scenario 2.

Table S14. Sensitivity analysis varying retirement ages in 2020

Scenario	Healthcare cost	Productivity loss	Societal cost
**(1) Male: 60 years Female: 55 years**	**US$ million [UI]**	**US$ million [UI]**	**US$ million [UI]**
All conditions	2,541 [1,408-4,083]	2,674 [1,574-4,128]	5,215 [2,982-8,211]
Cardiovascular diseases	842 [542-1,227]	984 [659-1,353]	1,826 [1,201-2,580]
Chronic respiratory diseases	51 [35-70]	8 [5-12]	59 [40-82]
Diabetes and kidney diseases	99 [65-141]	127 [79-195]	226 [143-336]
Digestive diseases	219 [114-341]	254 [148-356]	473 [262-697]
Maternal and neonatal disorders	39 [39-39]	16 [16-16]	55 [55-55]
Musculoskeletal disorders	44 [14-77]	42 [20-61]	86 [34-138]
Neoplasms	1,121 [544-1,974]	1,194 [605-2,074]	2,315 [1,149-4,049]
Neurological disorders	34 [10-75]	34 [29-45]	68 [39-120]
Sense organ diseases	79 [34-121]	3 [2-5]	82 [36-126]
Others	13 [11-16]	11 [11-12]	25 [22-28]
**(2) Male: 63 years Female: 58 years**			
All conditions	2,541 [1,408-4,083]	3,570 [2,101-5,517]	6,110 [3,509-9,599]
Cardiovascular diseases	842 [542-1,227]	1,292 [865-1,783]	2,134 [1,407-3,010]
Chronic respiratory diseases	51 [35-70]	13 [9-18]	64 [43-88]
Diabetes and kidney diseases	99 [65-141]	159 [99-243]	258 [163-383]
Digestive diseases	219 [114-341]	326 [190-457]	544 [305-798]
Maternal and neonatal disorders	39 [39-39]	18 [18-18]	57 [57-57]
Musculoskeletal disorders	44 [14-77]	54 [25-78]	98 [39-155]
Neoplasms	1,121 [544-1,974]	1,646 [843-2,839]	2,766 [1,387-4,813]
Neurological disorders	34 [10-75]	43 [35-60]	77 [45-135]
Sense organ diseases	79 [34-121]	4 [2-5]	82 [36-126]
Others	13 [11-16]	16 [15-16]	29 [26-32]

(3) Male: 65 years Female: 60 years	Healthcare cost<br>US$ million [UI]	Productivity loss<br>US$ million [UI]	Societal cost<br>US$ million [UI]
All conditions	2,541 [1,408-4,083]	4,031 [2,372-6,232]	6,572 [3,781-10,315]
Cardiovascular diseases	842 [542-1,227]	1,451 [971-2,004]	2,293 [1,513-3,232]
Chronic respiratory diseases	51 [35-70]	15 [10-21]	67 [45-92]
Diabetes and kidney diseases	99 [65-141]	175 [109-267]	274 [174-408]
Digestive diseases	219 [114-341]	363 [212-509]	581 [326-850]
Maternal and neonatal disorders	39 [39-39]	19 [19-19]	58 [58-58]
Musculoskeletal disorders	44 [14-77]	60 [28-87]	104 [42-164]
Neoplasms	1,121 [544-1,974]	1,878 [966-3,233]	2,999 [1,510-5,207]
Neurological disorders	34 [10-75]	48 [37-68]	81 [48-143]
Sense organ diseases	79 [34-121]	4 [2-6]	83 [36-127]
Others	13 [11-16]	18 [17-18]	31 [28-34]

US$, US dollar 2020 prices. UI, uncertainty interval.

Notes: Other conditions include mental disorders, nutritional deficiencies, other non-communicable diseases, self-harm and interpersonal violence, substance use disorders, unintentional injuries, enteric infections, HIV/AIDS and sexually transmitted infections, respiratory infections and tuberculosis, and other infectious diseases.

Attributable cost in year 2020 assuming individuals have no formal economic output (zero participation/salary) at ages above the retirement age. Scenario 1: Current retirement age of 60 years for males and 55 years for females. Scenario 2: Raising retirement age to 63 years for males and 58 years for females (policy reform goal for 2040). Scenario 3: Raising retirement age to 65 years for males and 60 years for females.
